# Steppe-Derived Glyphosate-Tolerant *Pseudomonas* sp. ADL-13 and *Paenibacillus* sp. ZHS-9 Strains with Plant Growth-Promoting Traits and Broad Abiotic Stress Tolerance

**DOI:** 10.3390/microorganisms14102371

**Published:** 2026-10-16

**Authors:** Adel Sattarova, Zhasmin Zhaksybek, Sándor Kocsubé, Irina Rukavitsina, Csaba Vágvölgyi, Sailau Abeldenov, Anuar Zhumakayev

**Affiliations:** 1 Laboratory of Molecular Biotechnology, National Center for Biotechnology, Astana 010000, Kazakhstan; 2 Department of Biotechnology and Microbiology, L.N. Gumilyov Eurasian National University, Astana 010000, Kazakhstan; 3 Department of Biotechnology and Microbiology, Faculty of Science and Informatics, University of Szeged, 6726 Szeged, Hungary; 4 Microbiology Laboratory, A.I. Barayev Research and Production Centre for Grain Farming, Nauchny Set., Akmola Region 021601, Kazakhstan

**Keywords:** *Pseudomonas* sp., *Paenibacillus* sp., glyphosate tolerance, PGPB, wheat growth-promotion, eco-physiological characterization, steppe environment

## Abstract

The integration of plant growth-promoting bacteria (PGPB) into sustainable agricultural practices represents a promising strategy for improving crop productivity and stress resilience. We characterized two newly isolated steppe-derived, glyphosate-tolerant bacteria, *Pseudomonas* sp. ADL-13 and *Paenibacillus* sp. ZHS-9, evaluating their in vitro glyphosate tolerance, PGP potential, and eco-physiological resilience. *Pseudomonas* sp. ADL-13 exhibited in vitro glyphosate tolerance, displaying significant growth stimulation at glyphosate concentrations of 1.0–2.0 g/L. *Paenibacillus* sp. ZHS-9 tolerated glyphosate in vitro up to 0.5 g/L without significant growth inhibition while maintaining measurable growth at 1.0 and 2.0 g/L. In wheat seedlings bioassays, cell-free culture filtrates promoted seedling growth, while inoculation with living cultures significantly enhanced biomass accumulation, seedling growth, and vigor. Plant growth-promoting (PGP) traits included P and K solubilization, and siderophore and ammonia production across strains; additionally, putative nitrogen-fixing potential, and IAA-like indolic compounds were detected in ZHS-9. Furthermore, both isolates demonstrated broad tolerance to saline, osmotic and oxidative stresses, pH and temperature fluctuations. Compatibility assays revealed antagonistic activity of ADL-13 toward ZHS-9, emphasizing the importance of evaluating strain interactions. Collectively, these findings indicate that ADL-13 and ZHS-9 are potential PGPB candidates for further evaluation in agricultural applications.

## 1. Introduction

Food security remains one of the major global challenges as agricultural systems face increasing pressure from population growth, land degradation, and climate change. Among agricultural practices aimed at maximizing crop production, herbicides remain indispensable for effective weed management, with glyphosate being the most extensively used herbicide worldwide. Glyphosate [N-(phosphonomethyl)glycine] is a systemic broad-spectrum herbicide widely applied before planting and during pre-harvest management in both conventional and herbicide-tolerant cropping systems [1]. Despite its agronomic benefits, its extensive and repeated use has raised concerns regarding the evolution of glyphosate-resistant weeds, the persistence of herbicide residues, and potential ecological and human-health effects [2,3,4,5,6,7]. Consequently, sustainable approaches that maintain crop productivity and mitigate chemical contamination while reducing environmental and human impacts are becoming increasingly important [8].

Soil microorganisms play fundamental roles in nutrient cycling, soil fertility, and ecosystem functioning, with the rhizosphere representing the primary interface where plants interact with beneficial microbial communities [9,10]. Plant growth-promoting bacteria (PGPB) enhance plant development through multiple direct and indirect mechanisms, including biological nitrogen fixation, phosphate and potassium solubilization, phytohormone and siderophore production, suppression of phytopathogens, and alleviation of abiotic stresses such as drought, salinity, temperature extremes, and oxidative stress [11,12,13,14]. Therefore, understanding and harnessing PGPB offers a sustainable strategy for mitigating these stresses and improving crop productivity while minimizing soil chemical contamination. The functional significance of PGPB becomes even more pronounced in glyphosate-contaminated soils, where herbicide exposure may impair plant growth and disturb rhizosphere microbial communities. Glyphosate accumulation has been associated with shifts in microbial community composition and increased abundance of phytopathogenic fungi; whereas, glyphosate-tolerant PGPB have been proposed as an effective biological strategy to support plant performance in agricultural systems exposed to herbicide usage [15,16].

Among beneficial PGPB, species of the genera *Pseudomonas* and *Paenibacillus* have attracted considerable attention because of their diverse plant growth-promoting capabilities. *Pseudomonas* species efficiently colonize plant roots and are well known for nutrient solubilization, phytohormone and siderophore production, and biological control of phytopathogens [17,18,19]. Likewise, *Paenibacillus* species are recognized for nitrogen fixation, phosphate and potassium solubilization, phytohormone production, and endospore formation, enabling survival under adverse environmental conditions [20,21]. These characteristics make both genera promising candidates for biofertilizer development in stressed agricultural environments.

Glyphosate-tolerant and plant growth-promoting bacteria, including strains from glyphosate-exposed agricultural soils in Kazakhstan, have been described previously [15,22,23]. However, comparative information integrating strain-specific glyphosate responses, baseline Plant growth-promoting (PGP) traits, plant responses to cell-free filtrates and living cells, broad abiotic-stress profiling, and direct compatibility testing remains limited for newly isolated steppe-derived strains from different bacterial genera. This remains insufficiently explored because native microbial communities inhabiting these steppe ecosystems are naturally exposed to pronounced seasonal temperature fluctuations and semi-arid conditions, making them a potentially valuable yet underexplored source of environmentally resilient plant growth-promoting bacteria. Therefore, the present study asked whether newly isolated glyphosate-tolerant bacteria from agricultural steppe soils can combine baseline plant growth-promoting functions with tolerance to multiple abiotic stresses under laboratory conditions. Two newly isolated strains, *Pseudomonas* sp. ADL-13 and *Paenibacillus* sp. ZHS-9, were comprehensively characterized for their glyphosate tolerance, baseline plant growth-promoting effects on early wheat seedling development and PGP mechanisms, followed by an assessment of eco-physiological tolerance, compatibility, and cellular morphology. This study provides a foundational characterization of resilient steppe-derived isolates with potential applications in sustainable agriculture.

## 2. Materials and Methods

### 2.1. Isolation and Identification of Indigenous Glyphosate-Tolerant Bacteria

#### 2.1.1. Isolation of Indigenous Glyphosate-Tolerant Bacteria

A previously described protocol [23] was adapted to isolate indigenous glyphosate-tolerant bacteria from agricultural steppe soils in Kazakhstan. Two individual samples of bulk soil (SW-1 and SW-3) were collected in August 2024 from two distinct agricultural field sites cultivated with spring soft wheat (*Triticum aestivum* L.) in the Akmola region of northern Kazakhstan (sampling depth: 0–20 cm). Sample SW-1 was collected from a Calcaric Haplic Chernozem field cultivated with cultivar “Astana”, whereas SW-3 was collected from a Haplic Chernozem field cultivated with cultivar “Element 22”. Although specific soil physicochemical parameters were not determined experimentally in this study, both fields had a documented history of glyphosate-based herbicide application. For SW-1, the field had received “Uragan Forte” at 2 L/ha as a pre-sowing chemical treatment in May 2023 and for chemical weeding of no-till fallow in August 2023, followed by “Pharaon Gold” at 2 L/ha as a pre-sowing treatment in May 2024. For SW-3, “Smerch” was applied at 2 L/ha as a pre-sowing chemical treatment in June 2024. Further information on the soil samples and crop history is provided in Supplementary Table S1.

Both direct selective plating and enrichment culture were employed to maximize the recovery of phylogenetically and physiologically diverse glyphosate-tolerant bacteria. A soil suspension was prepared by homogenizing 5 g of bulk soil sample in 40 mL of sterile 0.9% NaCl solution. One milliliter of soil suspension was serially tenfold diluted in three steps (10^−1^, 10^−2^, and 10^−3^) and 50 µL from each dilution step was plated on solid *Pseudomonas* minimal medium (PMM, [24], containing (g/L): (NH_4_)_2_SO_4_, 4.49; K_2_HPO_4_, 1.5; MgSO_4_·7H_2_O, 0.2; agarose, 20. PMM was supplemented with the commercial glyphosate-based herbicide “Uragan Forte” (500 g/L glyphosate acid equivalent as potassium salt, Syngenta). Unless stated otherwise, all glyphosate concentrations reported in this study are expressed as g/L glyphosate acid equivalent (a.e.) delivered through the commercial formulation. PMM was supplemented at 1 g/L as the principal selective agent to isolate glyphosate-tolerant bacterial strains, alongside nystatin (0.05 g/L) and fluconazole (0.05 g/L) as fungicides to prevent fungal growth. For enrichment culture method, 1 mL of soil suspension was inoculated in liquid PMM amended with glyphosate and fungicides as above and incubated at 25 °C, 150 rpm on a rotary shaker IKA KS 260, Staufen im Breisgau, Germany). After 3 days of incubation, 1 mL of the enrichment culture was transferred into fresh PMM containing glyphosate and antifungal compounds at the same concentrations. This enrichment step was repeated once more under identical conditions. One milliliter of the final enrichment culture was used to inoculate solid PMM supplemented with glyphosate and fungicides at the same concentrations described above. The plates were incubated at 25 °C for 7 days in a standard incubator BD-56 (Binder GmbH, Tuttlingen, Germany). The grown individual colonies were attributed as glyphosate-tolerant isolates and purified on yeast-extract–glucose medium (YEG, g/L: yeast extract, 5; glucose, 10; agar, 15).

#### 2.1.2. Identification of Steppe-Derived Glyphosate-Tolerant Bacteria

Purified glyphosate-tolerant bacterial isolates were identified by 16S rRNA gene sequencing to determine taxonomic affiliation and identify close relatives with known pathogenic characteristics. Universal bacterial primers Eub341F (5′-CCTACGGGAGGCAGCAG-3′) and Eub1060R (5′-CGACACGAGCTGACGACA-3′) were used to amplify a partial 16S rRNA gene fragment [25]. DNA template was prepared using overnight-grown bacterial cultures adjusted in sterile distilled H_2_O at 10^7^ CFU/mL. The PCR reaction mixture (50 μL total volume), contained 2 μL each of the 10 μM forward and reverse primers, 10 μL of 5X HF buffer, 1 μL of 10 mM dNTP mix, 1 μL of Phusion DNA polymerase, 1 μL DNA template, and 33 μL sterile distilled H_2_O. PCR was performed in a T100 Thermal Cycler (Bio-Rad, Singapore) under the following conditions: initial denaturation at 95 °C for 2 min; 32 cycles of 95 °C for 30 s, 57 °C for 45 s, and 72 °C for 1 min; followed by a final extension at 72 °C for 7 min. PCR products were run in 1% agarose gel electrophoresis for 40 min at 100 V and analyzed using a GelDoc Go Imaging System (Bio-Rad, USA). PCR products were treated with ExoI and FastAP followed by BigDye-based sequencing reaction (ThermoFisher Scientific Baltics UAB, Vilnius, Lithuania). The obtained amplicons were purified using an ethanol/ethyl acetate precipitation followed by resuspension in formamide and submission for Sanger sequencing to an external service (National Scientific Shared Laboratory of Biotechnology, National Center for Biotechnology, Astana, Kazakhstan). The obtained sequences were subjected to NCBI BLAST analysis to identify genera and species of the glyphosate-tolerant strains. In addition, partial sequences of *rpoB* and *rpoD* [26] from ADL-13, and *gyrA1* and *gyrA2* [22] from ZHS-9, were obtained using the corresponding primers and PCR programs and used for strain identification.

Based on genus-level identification, all isolates were classified into three genera: *Paenarthrobacter*, *Paenibacillus*, and *Pseudomonas*. All obtained sequences were deposited in the NCBI database under the accession numbers listed in Table 1.

For the phylogenetic analysis of the isolates, sequences of closely related species were retrieved from the NCBI Datasets database (Supplementary Table S2). For the *Paenibacillus* sp. isolate, the 16S rRNA and *gyrA* sequences were included in the analysis, while in the case of the *Pseudomonas* sp. isolate, the 16S rRNA, *rpoB*, and *rpoD* gene sequences were analyzed. Multiple sequence alignments were performed using the sequences of the isolates together with those of closely related species in MAFFT version 7.526 [27]. Alignments were generated using the G-INS-i algorithm. For both groups (*Paenibacillus* and *Pseudomonas*) the resulting alignments were manually trimmed to an equal length and concatenated into a single dataset using SequenceMatrix version 1.8 [28]. The concatenated *Paenibacillus* and *Pseudomonas* datasets consisted of 1700 and 1590 characters, respectively, of which 249 and 155 characters were parsimony-informative. The concatenated dataset was partitioned, and evolutionary model selection was performed using the built-in ModelFinder algorithm [29] implemented in IQ-TREE. Phylogenetic reconstruction was then carried out using IQ-TREE v 2.0.7 [30] by applying the best-fitting evolutionary model to each partition. Statistical support of the best-scoring phylogenetic tree was assessed using the ultrafast bootstrap (UFBoot) [31] algorithm with 5000 replicates.

### 2.2. Evaluation of Glyphosate Tolerance

#### 2.2.1. Solid Medium Assay

Glyphosate tolerance of the purified bacterial isolates was initially evaluated on solid PMM supplemented with 0.1, 0.5, and 1.0 g/L of glyphosate (a.e.) using the commercial glyphosate-based herbicide (“Uragan Forte”), as described above. Overnight-grown bacterial isolates were spot-inoculated on PMM plates, and their colony diameter was measured after 7 days of incubation at 25 °C. All assays were performed using three independent biological replicates (*n* = 3).

#### 2.2.2. Liquid Medium Assay

Broth microdilution assay was applied to examine glyphosate tolerance of bacterial isolates in liquid cultures using YEG supplemented with a broad range of glyphosate concentrations (a.e.: 0.05, 0.1, 0.2, 0.5, 1.0, 2.0, and 5.0 g/L) supplied through a commercial formulation as above. Bacterial cultures pre-grown on solid YEG for 24 h were collected in 5 mL 0.9% NaCl and used to prepare cell suspensions adjusted to 10^6^ CFU/mL in 0.9% NaCl. Twenty μL was inoculated into 96-well Nunc MicroWell 96-Well Microplate (Thermo Fisher Scientific, USA) containing 180 μL of glyphosate-amended YEG, resulting in an initial cell density of 10^5^ CFU/mL. Inoculated YEG without glyphosate served as the control, while non-inoculated YEG with adjusted glyphosate concentrations was used as blank samples to verify the absence of an effect of glyphosate amendments on OD measurements. Plates were incubated at 25 °C at 200 rpm using a CAPP Rondo Place Incubating Shaker, CRPI-412X (AHN Biotechnologie GmbH, Nordhausen, Germany). Bacterial growth was monitored by measuring optical density at 620 nm (OD_620_) after 48 h using a Multiskan SkyHigh Microplate Spectrophotometer (Thermo Fisher Scientific, Singapore). The Multiskan SkyHigh Microplate Spectrophotometer was used for all optical density measurements throughout the study, including those performed at different wavelengths in subsequent assays. All assays were performed in triplicate.

### 2.3. Assessments of Plant Growth Promotion Potential

#### 2.3.1. The Effect of Bacterial CCF on Wheat Seedling Growth

Initial assessment of plant growth-promoting potential of glyphosate-tolerant bacterial strains was performed using a wheat seedling bioassay with cell-free culture filtrates (CCFs). CCFs were obtained from liquid cultures grown in 50 mL Minimal Medium (MM; g/L: KH_2_PO_4_, 1.0; MgSO_4_·7H_2_O, 0.5; (NH_4_)_2_SO_4_, 5.0, [32] supplemented with 2 g/L L-alanine (for *Pseudomonas* and *Paenarthrobacter* strains), [33] and in 50 mL YEG (for *Paenibacillus* strains because these strains were unable to grow in MM under all tested conditions). Both MM and YEG were amended with 0.1 g/L L-tryptophan (VWR) as a precursor to induce indole-3-acetic acid (IAA) production. Bacterial suspensions prepared in 5 mL 0.9% NaCl were used to inoculate the respective amended MM and YEG at 10^5^ CFU/mL initial cell density and these were incubated at 130 rpm, 25 °C for 3 days. After incubation, cultures were centrifuged at 6000× g for 10 min, and the supernatants were filter-sterilized through 0.22 µm syringe filters (TPP, Schaffhausen, Switzerland) to obtain sterile CCF. Sterility of the CCFs was confirmed by incubating 5 µL on YEG agar plates at 25 °C for 3 days. Non-inoculated MM or YEG treated identically to the bacterial cultures were used as the controls to evaluate the effects of the culture media.

The effect of the obtained bacterial CCF on the plant growth characteristics was evaluated on soft spring wheat seedlings (cultivar “Akmola-2”) in pot assays. In a pot experiment, 100 wheat seeds were soaked in 10 mL of corresponding CCF for 2 h and 10 randomly selected seeds were planted in 250 mL pots filled with commercially available soil substrate (neutralized multi-purpose sphagnum peat-moss substrate supplemented with mineral macro- and micronutrients; pH ≥ 5.5) under a completely randomized design (CRD). Seeds soaked in distilled water (WC) or non-inoculated CCF (MC-I for MM amended with L-alanine and MC-II for YEG) served as the controls to compare the effect of bacterial CCFs with dH_2_O and to distinguish bacterial CCF effects from sterile medium components, respectively. Additionally, on days 3 and 5 after sowing, 5 mL of CCF was applied on bacterial-treated samples while WC and corresponding MC-I/MC-II received 5 mL of distilled water or 5 mL of non-inoculated CCF, accordingly. All treatments were also irrigated with 5 mL distilled water immediately upon planting and subsequently on the 3rd and 5th days of incubation.

The plants were grown for 7 days under natural day–night cycle at 25 °C. The fresh mass, number of roots, seedling length, root length, total length, and vigor index were measured to evaluate the plant growth-promoting potential of glyphosate-tolerant strains. Germination rate was computed as the number of germinated seeds divided by the total number of total planted seeds, multiplied by 100. Total length was calculated as the sum of seedling and root lengths. The vigor index was calculated as the total length multiplied by the germination rate. The percentage increase in each growth parameter relative to the corresponding control was calculated as (treatment—control)/control, multiplied by 100. Only germinated seeds were used for all analyses [34] to verify the bacterial effect on the plant growth. Each treatment consisted of three biological replicates, with the individual pot (containing 10 seeds) serving as the experimental unit. Measurements from individual seedlings within a pot were averaged to represent a single biological replicate value prior to statistical analysis.

Based on the complementary criteria, glyphosate tolerance and plant growth responses, two strains, *Pseudomonas* sp. ADL-13 and *Paenibacillus* sp. ZHS-9, were selected for subsequent assays. ADL-13 showed the strongest combined profile among the *Pseudomonas* isolates; whereas, ZHS-9 was selected among the *Paenibacillus* isolates because it retained the strongest glyphosate-tolerance profile in that group. Both strains showed positive CCF-associated growth trends. These strains were consequently studied in all subsequent assays.

#### 2.3.2. The Effect of Bacterial Living Cultures on Wheat Seedling Growth

The effect of the selected living bacterial cultures of *Pseudomonas* sp. ADL-13 and *Paenibacillus* sp. ZHS-9 on wheat growth was studied in an independent pot assay using seeds of the same cultivar under a distinct CRD. Strain ADL-13 was cultivated in MM supplemented with 2 g/L L-alanine, whereas strain ZHS-9 was grown in YEG; both media were supplemented with 0.1 g/L L-tryptophan. All cultures inoculated at an initial cell density of 10^5^ CFU/mL were incubated at 150 rpm, 25 °C for 3 days. The seed soaking, irrigation, incubation conditions, and incubation-stage treatments, including control treatments (WC, MC-I, and MC-II) correspond identically to the previous section, except that fresh three-day-old liquid bacterial cultures were applied in each treatment, instead of CCF. Plant growth parameters were recorded upon 7-day incubation and analyzed as described above.

### 2.4. Plant Growth-Promoting Characteristics

#### 2.4.1. Putative Nitrogen-Fixing Capability

Putative nitrogen-fixing potential was screened using Ashby’s nitrogen-free agar medium (g/L: glucose, 20; K_2_HPO_4_, 0.2; MgSO_4_, 0.2; NaCl, 0.2; K_2_SO_4_, 0.1; CaCO_3_, 5.0; agar, 15.0). The pH was adjusted to 7.4 using 1 M NaOH before sterilization. Ashby’s medium supplemented with 0.27 g/L NH_4_Cl as a nitrogen source served as the control. Bacterial suspensions prepared from overnight-grown cultures were adjusted to 10^7^ CFU/mL in 5 mL 0.9% NaCl, and 5 µL were spot-inoculated on Ashby’s agar plates. The plates were incubated at 25 °C for 7 days and the colony diameter was measured on the 3rd, 5th, and 7th days of incubation. Consistent bacterial growth on nitrogen-free medium throughout the three observation time points was considered indicative of putative nitrogen fixation capability.

#### 2.4.2. Phosphorus Solubilization Activity

Phosphate solubilization activity of bacterial isolates was evaluated using Pikovskaya agar medium [35] (g/L: glucose, 10.0; (NH_4_)_2_SO_4_, 0.5; MgSO_4_·7H_2_O, 0.1; KCl, 0.2; yeast extract, 0.5; NaCl, 0.2; MnSO_4_·H_2_O, 0.002; FeSO_4_·7H_2_O, 0.002; agar, 20.0). Tricalcium phosphate (Ca_3_(PO_4_)_2_) and dicalcium phosphate (CaHPO_4_) [36] were evaluated separately at 5 g/L as insoluble phosphorus sources. Bacterial suspensions prepared from overnight cultures were adjusted to a final concentration of 10^7^ CFU/mL, and a 10 µL aliquot of the suspension was spot-inoculated onto the agar surface, and air-dried for 10 min. Plates were incubated at 25 °C for 10 days and the colony diameter and total diameter (measured from outer edge to outer edge of the clear zone, encompassing the colony) were measured in millimeters (mm). Phosphate solubilization activity was evaluated based on the formation of clear halo zones around bacterial colonies. The phosphorus solubilization index (PSI) was calculated using the following formula:

(1) $$\mathrm{PSI}=\frac{\mathrm{total}\;\mathrm{diameter}}{\mathrm{colony}\;\mathrm{diameter}}$$

#### 2.4.3. Potassium Solubilization Activity

The ability of bacterial isolates to solubilize potassium was examined using commercial Aleksandrow medium containing 2 g/L potassium alumina silicate (HiMedia, India). Bacterial suspension, inoculation, incubation, and measurements were performed as described for phosphorus solubilization assays above. The formation of clear halo zones around bacterial colonies was considered indicative of potassium solubilization activity. The potassium solubilization index (KSI) was calculated using the same equation applied for PSI:

(2) $$\mathrm{KSI}=\frac{\mathrm{total}\;\mathrm{diameter}}{\mathrm{colony}\;\mathrm{diameter}}$$

#### 2.4.4. Synthesis of IAA-like Indolic Compounds

For testing IAA-like indolic compounds production, bacterial strains were inoculated in 30 mL YEG supplemented with 0.1 g/L L-tryptophan (VWR, Leuven, Belgium) at an initial cell density of 10^5^ CFU/mL. The strains were cultivated at 130 rpm, 25 °C, and samples were collected after 5 days of incubation. The samples were centrifuged at 10,000× *g* for 10 min and 100 µL of the obtained supernatant was mixed with 100 µL of Salkowski reagent (12 g/L FeCl_3_·6H_2_O in 37% H_2_SO_4_) [37]. The mixture was incubated at 25 °C in the dark for 20 min and 200 µL of the reaction mixture was measured spectrophotometrically at 530 nm (OD_530_). The obtained OD values were analyzed for IAA concentration based on a standard curve generated using a stock solution of IAA standard (Thermo Scientific) dissolved in DMSO. Serial dilutions ranging from 0 to 500 µg/mL (0, 50, 60, 70, 80, 90, 100, 200, 300, 400, and 500 µg/mL IAA in DMSO) were prepared in triplicate to construct a calibration curve. The calibration curve was generated by linear regression of the mean absorbance values using SkanIt RE 7.0.2 software (provided by the Multiskan SkyHigh spectrophotometer’s manufacturer) and applied to quantify IAA concentrations in the culture supernatants.

#### 2.4.5. Siderophore Production

Siderophore production was evaluated using the Chrome Azurol S (CAS) colorimetric assay [38]. The CAS complex, containing a mixture of three solutions, was prepared as follows: 10 mL of 1 mM FeCl_3_·6H_2_O in 10 mM HCl was added to 50 mL of 2 mM CAS solution, followed by the addition of 40 mL of 5 mM hexadecyltrimethylammonium bromide (HDTMA) in distilled water. Iron-depleted standard succinate medium [39] (SMM, g/L: K_2_HPO_4_, 6.0; KH_2_PO_4_, 3.0; (NH_4_)_2_SO_4_, 1.0; MgSO_4_·7H_2_O, 0.2) was supplemented with 4 g/L glucose (SMM + Glucose) or succinic acid (SMM + Succinic acid) to stimulate siderophore synthesis, individually. Additionally, siderophore production was studied in YEG because *Paenibacillus* sp. ZHS-9 was unable to grow in MM. Bacterial cultures inoculated at 10^5^ CFU/mL as an initial cell density were incubated at 130 rpm, 25 °C for 3 days. Non-inoculated MM or YEG treated identically to the bacterial cultures served as the reference samples. The culture supernatant was collected by centrifugation at 10,000× *g* for 10 min and a 50 µL sample was mixed with 150 µL of CAS complex. The mixture was incubated at 25 °C for 20 min. The color change in the reaction indicated chelation of Fe^3+^ ions from the CAS complex by bacterial siderophores. The absorbance of the reaction was measured at OD_630_ and percentage siderophore units (PSU) was calculated according to the equation by Payne [40]:

(3) $$\mathrm{PSU},\%=\frac{\mathrm{reference}\;\mathrm{sample}+\mathrm{inoculated}\;\mathrm{sample}}{\mathrm{reference}\;\mathrm{sample}}\times 100$$

#### 2.4.6. Ammonia Production

Ammonia (NH_3_) production was quantified using the indophenol blue colorimetric method. Bacterial strains were inoculated into YEG and commercial peptone water (PW; Titan Biotech, India) at 10^5^ CFU/mL as an initial density. Cultures were incubated at 25 °C, 130 rpm for 3 (YEG) and 7 days (peptone water) to account for the different bacterial growth in these two media, and samples were taken on the relevant final day of incubation. Non-inoculated peptone water and YEG served as the control. For ammonia determination, 2 mL of culture was centrifuged at 10,000× *g* for 10 min and 100 µL of the supernatant was mixed with 200 µL phenol-based reagent and subsequently with 200 µL alkaline hypochlorite reagent. The mixture was briefly vortexed and incubated at 37 °C for 20 min in a water bath (WB-4MS, BioSan, Latvia). The development of blue color at the end of incubation was attributed to NH_3_ production and absorbance was measured at 540 nm (OD_540_). The recorded OD_540_ values were converted into quantitative NH_3_ concentrations based on a calibration curve prepared using ammonium sulfate (NH_4_)_2_SO_4_) over the range of 0, 10, 20, 30, 40, 50, 60, 70, 80, 90, and 100 µM prepared in dH_2_O. Standards were processed under the same conditions as described for the bacteria-derived samples and the calibration curve was constructed by plotting absorbance values against standard concentrations using SkanIt software. The generated calibration curve was used for the quantification of NH_3_ concentrations in the bacterial samples.

#### 2.4.7. Organic Acid Secretion

Organic acid (OA) production by bacterial isolates was evaluated using a modified Ashby’s medium (described in Section 2.4.1), supplemented with 5 g/L CaCO_3_ [41], 5 g/L yeast extract, and 0.27 g/L NH_4_Cl. The inoculation procedure corresponded to the phosphorus and potassium solubilization assays (Section 2.4.2 and Section 2.4.3), and the plates were incubated at 25 °C for 7 days. The formation of clear halo zones around colonies indicated organic acid-mediated CaCO_3_ solubilization.

#### 2.4.8. Biofilm Formation

Biofilm formation was quantified using the crystal violet microtiter plate assay [42]. Overnight bacterial cultures grown on YEG were harvested in 0.9% NaCl and adjusted to 10^8^ CFU/mL. For initial adhesion, 200 µL of the prepared bacterial suspension was added to wells of a 96-well microplate, while wells containing non-inoculated YEG served as the controls. Plates were incubated at 25 °C for 4 h under static conditions to allow cell adherence, then the cultures were gently removed. Non-adherent cells were removed by washing the wells three times with 0.9% NaCl. Subsequently, 200 µL of fresh YEG medium was added to each well, and the plates were incubated statically at 25 °C for 24 h to allow biofilm development. After incubation, the planktonic cultures were removed, the wells were gently washed three times with 0.9% NaCl, and the attached biofilms were fixed with methanol (MeOH) for 15 min at 25 °C. Then, MeOH was removed and the plate was air-dried for 10 min. Biofilm was stained with 0.1% crystal violet (Thermo Scientific) for 20 min at 25 °C, rinsed thoroughly with dH_2_O, and air-dried for 20 min. The bound dye was solubilized with 33% acetic acid, and biofilm formation was quantified by measuring OD_590_. Biofilm formation was evaluated by comparing OD_590_ values of the inoculated samples with identically treated non-inoculated controls. Higher OD_590_ values reflected increased crystal violet retained by the attached biofilm and indicated greater biofilm-forming capacity. The test was performed using six biological replicates (*n* = 6).

### 2.5. Eco-Physiological Profiling of Steppe-Derived Pseudomonas sp. ADL-13 and Paenibacillus sp. ZHS-9

#### 2.5.1. Tolerance to Abiotic Stress Factors

The eco-physiological profile of *Pseudomonas* sp. ADL-13 and *Paenibacillus* sp. ZHS-9 was characterized by evaluating their tolerance to salinity, drought, surfactant Tween 80, short-term oxidative stress, and by determining their optimal pH and temperature ranges. To ensure consistency across all eco-physiological assays, the protocol described for salinity tolerance (including bacterial suspension preparation, initial cell density, inoculation, incubation, controls, and OD measurements) was applied to all relevant assays. Inoculated YEG without the tested stress factor served as the control (Level 1) to compare with bacterial growth under increasing stress levels (Levels 2–8). Non-inoculated YEG with the corresponding concentrations of each stress factor served as the control for background OD measurements. All treatments were performed using three independent biological replicates (*n* = 3).

*Salinity tolerance.* Salinity tolerance of the isolates was assessed by cultivating bacterial strains in 96-well microplates containing 180 µL liquid YEG supplemented with different NaCl concentrations (0, 2, 5, 10, 15, 20, 25, and 50 g/L for Levels 1–8, respectively). Bacterial cultures pre-grown on solid YEG for 16 h at 25 °C were collected in 0.9% NaCl using cotton swabs and 200 µL of the obtained cell suspension were subjected to OD_620_ measurements to quantify cell density. Based on the obtained OD-values, the subsequent bacterial suspension was prepared in 0.9% NaCl and adjusted to 10^6^ CFU/mL. Twenty µL from this suspension were transferred in the wells of a Nunc™ MicroWell™ 96-well microplate, containing 180 µL of NaCl-amended YEG medium, resulting in a final cell density of 10^5^ CFU/mL. The plates were incubated at 25 °C at 200 rpm in CAPP Rondo Place Incubating Shaker, CRPI-412X. Bacterial growth was determined by measuring optical density at 620 nm (OD_620_), after 48 h of incubation.

*Drought tolerance.* Drought tolerance was assessed by evaluating growth under osmotic stress conditions simulated with polyethylene glycol 6000 (PEG 6000) [43]. YEG was supplemented with PEG 6000 at final concentrations of 0, 25, 50, 100, 150, 200, 250, and 300 g/L for Levels 1–8, respectively.

*Oxidative stress tolerance.* Short-term oxidative burst was mimicked via the addition of H_2_O_2_ in liquid YEG to induce reactive oxygen species (ROS)-mediated oxidative stress. This design attempts to model a short-term oxidative burst—analogous to the transient ROS challenges that plant-associated bacteria may encounter in during abiotic stress episodes and consistent with previous methodologies used to evaluate oxidative tolerance in beneficial rhizobacteria [44]. Prior to YEG amendments, seven stocks of H_2_O_2_ were prepared in sterile distilled water in the concentrations as 0.4, 2.0, 4.0, 8.0, 12.0, 20.0, and 40.0 mM. From each stock, 5 µL was transferred to a 96-well plate containing 175 µL YEG to obtain the final tested concentrations of 0.01, 0.05, 0.1, 0.2, 0.3, 0.5, and 1.0 mM for Levels 2–8, respectively.

*Surfactant tolerance.* Tolerance of glyphosate-tolerant strains to the presence of Tween 80 was studied in 96-well microplates [45] containing Tween-amended YEG with final concentrations, v/v as 0.010, 0.025, 0.05, 0.1, 0.25, 0.5, 1.0%, for Levels 2–8, respectively.

*Optimal temperature range.* The effect of temperature on the bacterial growth was examined at different incubation temperatures (10, 15, 20, 25, 30, 35, 40, and 45 °C for Level 1–8, respectively) using bacterial suspensions prepared from cultures pre-grown at 25 °C as described above.

*Optimal pH range.* The pH range of bacterial growth was evaluated in YEG medium adjusted to different pH values using Britton–Robinson buffer [46], consisting of Buffer A and Buffer B. Buffer A was prepared by mixing CH_3_COOH, H_3_PO_4_, and H_3_BO_3_ (each 0.04 M) and 100 mL of Buffer A was titrated with Buffer B (0.2 M NaOH) to obtain final pH values of 5.21, 6.38, 7.23, 7.85, 8.51, 9.21, 9.98, and 10.61, for Levels 1–8, respectively. Final pH was verified with a SevenDirect SD20 pH meter (Mettler Toledo, China). The obtained buffers were sterilized with 0.22 µm membrane filters and mixed in equal proportions with YEG, prior to inoculation.

#### 2.5.2. Heat and Cold Shock Assay

The ability to resume growth following heat and cold shock was evaluated using a stepwise temperature exposure assay. Bacterial suspensions were prepared in 0.9% NaCl and adjusted to a final cell density of 10^7^ CFU/mL. Aliquots (300 µL) were exposed to −20, −10, 0, 5, 25, 40, 50, and 60 °C for 5, 30, or 60 min, corresponding to short-, medium-, and long-term exposure, respectively. Following temperature–time exposure, samples were immediately removed from the respective treatment temperatures and allowed to return to room temperature within 5–10 min. Then, 100 µL of each treated suspension was transferred into wells of a 96-well microplate containing 100 µL of sterile double-concentrated YEG medium. The originally prepared suspension maintained at 25 °C throughout the assay represented the incubation temperature employed in all other assays described above. Plates were incubated at 25 °C at 200 rpm for 24 h and bacterial growth was measured at optical density 620 nm (OD_620_). This assay enabled comparison of bacterial recovery following heat and cold shock treatments at different exposure times. All treatments were carried out in three replicates.

#### 2.5.3. Qualitative Compatibility Assays

Before consortium formation between *Pseudomonas* sp. ADL−13 and *Paenibacillus* sp. ZHS-9, both strains were co-cultured using agar plate assays to verify mutual compatibility. Fresh 24 h cultures grown on solid YEG were collected in 0.9% NaCl with cotton swabs and used to prepare bacterial suspensions in 0.9% NaCl at cell densities of 10^5^, 10^6^, and 10^7^ CFU/mL. Five mL of the prepared suspension at 10^5^ CFU/mL were used to uniformly flood the surface of YEG agar plates. Excess suspension was removed, and plates were air-dried for 15 min. Subsequently, 10 µL aliquots of the tested strain suspensions (three concentrations per plate) were spot-inoculated onto the agar surface. Plates were air-dried for an additional 10 min prior to incubation. The assay was performed reciprocally by alternating each isolate as the lawn and the spot inoculant. The plates were incubated at 25 °C for 3 days. Antagonistic activity was assessed based on the presence of visible inhibition zones surrounding the spot-inoculated colonies. All assays were performed in triplicate.

#### 2.5.4. Scanning and Transmission Electron Microscopy

Scanning electron microscopy (SEM) and transmission electron microscopy (TEM) were performed to examine the cellular morphology and surface architecture of bacterial strains ADL-13 and ZHS-9 grown under standard cultivation conditions. Bacterial cultures were grown in liquid YEG medium for 24 h at 25 °C and 130 rpm with an initial cell density of 10^5^ CFU/mL. After incubation, 1 mL of bacterial culture was fixed in 2.5% glutaraldehyde prepared in phosphate buffer (pH 7.2–7.4) at 4 °C for 24 h. The fixed samples were submitted for SEM and TEM analyses to an external service (Electron Microscopy Laboratory, Core Facilities and HPC, Nazarbayev University).

Samples were washed with the same buffer and post-fixed in 1% osmium tetroxide for approximately 1 h, followed by dehydration through a graded ethanol series (30–100%). For SEM analysis (Auriga Crossbeam 540, Carl Zeiss, Germany), dehydrated samples were placed onto collagen-coated coverslips, air-dried, mounted on holders, and coated with a thin conductive carbon layer prior to imaging using an Auriga Crossbeam 540. For TEM analysis (JEM 1400 Plus, JEOL, Japan), dehydrated samples were infiltrated with epoxy resin and polymerized at 60 °C. Ultrathin sections (60–90 nm) were prepared using a Leica UC7 ultramicrotome (Leica Microsystems, Germany) and examined with a JEM 1400 Plus.

### 2.6. Statistical Analyses and Data Visualization

For the concentration–response assays evaluating cell growth under glyphosate, NaCl, PEG 6000, Tween 80, and ROS exposure, statistical comparisons were performed separately for each bacterial strain, with treatment concentration as the categorical factor. Thus, each strain–stress factor combination was analyzed independently using one-way Analysis of Variance (ANOVA) followed by Tukey’s HSD post hoc comparisons to identify differences among concentrations. Prior to ANOVA, the assumptions of normality and homoscedasticity were assessed. Normality of the residuals was evaluated using visual inspection of Q-Q plots and the Shapiro–Wilk test, while homoscedasticity was assessed using Levene’s test. Given the limited power of formal assumption tests at small sample sizes (*n* = 3), graphical assessment of the data and residuals was also considered when evaluating model assumptions. In cases where the data did not meet the required assumptions, a non-parametric alternative for ANOVA, the Kruskal–Wallis test, was applied. Significant differences were indicated by different letters, with statistical significance accepted at α = 0.05 (*p* < 0.05).

To evaluate the growth-promoting effects of bacterial strains in comparison to the controls (WC, MC-I/MC-II), pot mean values (*n* = 3) were subjected to one-way ANOVA with treatment as the factor, followed by Dunnett’s post hoc test at a significance level of *p* < 0.05. Dunnett’s test was applied to compare each bacterial treatment with WC or corresponding MC-I/MC-II, individually. This test was selected instead of Tukey’s HSD, as the primary aim of the study was to compare each treatment against the controls rather than performing all pairwise comparisons. Prior to analysis, assumptions were checked as described above and since the required assumptions were confirmed, parametric ANOVA was used. The significant difference from the controls was denoted as ns: non-significant, *p* > 0.05, * *p* < 0.05; ** *p* < 0.01; *** *p* < 0.001.

For the biofilm formation assay, OD_590_ values of ADL-13, ZHS-9, and the control were compared using one-way ANOVA followed by Tukey’s HSD post hoc test (*n* = 6). The assumptions were checked as described above. Significant differences were indicated by different letters, with *p* < 0.05 considered statistically significant.

To characterize optimal pH and temperature range as well as growth recovery trends after exposure to heat- and cold-shock assays, descriptive statistics such as mean and standard deviations (SD) were applied, since these experiments were intended for descriptive characterization rather than formal hypothesis testing.

All statistical analyses and data visualization were performed in R 4.4.1 programming language (https://www.r-project.org/ (accessed on 12 October 2026)) and RStudio 2024.04.2 (https://posit.co/ (accessed on 12 October 2026)) software. Levene’s and Dunnett’s tests were run using the packages *car* and *DescTools* [47,48], respectively. The *agricolae* package [49] was applied to generate letter groupings of Tukey’s HSD multiple comparisons. All graphs were constructed using the *ggplot2* package [50] and merged using the *patchwork* package [51]. Unless otherwise specified, experiments were performed using three independent biological replicates (*n* = 3). The data are presented as mean ± SD, unless otherwise specified.

## 3. Results

### 3.1. Isolation and Identification of Glyphosate-Tolerant Strains from the Steppe Environment

In total, 10 glyphosate-tolerant strains were obtained from soil samples collected from underexplored steppe environments. Serial dilution and plating on the PMM selective medium supplemented with 1 g/L glyphosate yielded morphologically distinct colonies (Table 1).

Sequencing of the 16S rRNA gene and subsequent BLAST analysis enabled molecular identification of the isolates at the genus level. The isolates ZHS-4, ZHS-5, and ZHS-6 were identified as *Paenarthrobacter* sp. whereas isolates ZHS-8, ZHS-9, AZ-1, AZ-2, and AZ-3, as well as ADL-13 and ADL-20, were identified as *Paenibacillus* and *Pseudomonas* sp., respectively.

All isolates, regardless of species, showed strain-dependent growth responses across the tested glyphosate concentrations on solid agar plate assays. Colony diameter varied from the minimum of 2.00 to the maximum of 4.57 mm for *Pseudomonas* sp. ADL-20 at 1.0 g/L and *Paenarthrobacter* sp. ZHS-6 at 0.1 g/L, respectively. The three strains of *Paenarthrobacter* grew at 3.00–4.57 mm range, depending on the strain and glyphosate concentration. Regarding the five isolated *Paenibacillus* strains, the highest colony diameter was recorded for the AZ-1 isolate, whereas in the two *Pseudomonas* isolates, the highest growth was observed for ADL-13. Overall, the data suggest in vitro glyphosate tolerance of the steppe-derived strains since growth was observed across the full concentration range tested.

**Figure 1:**
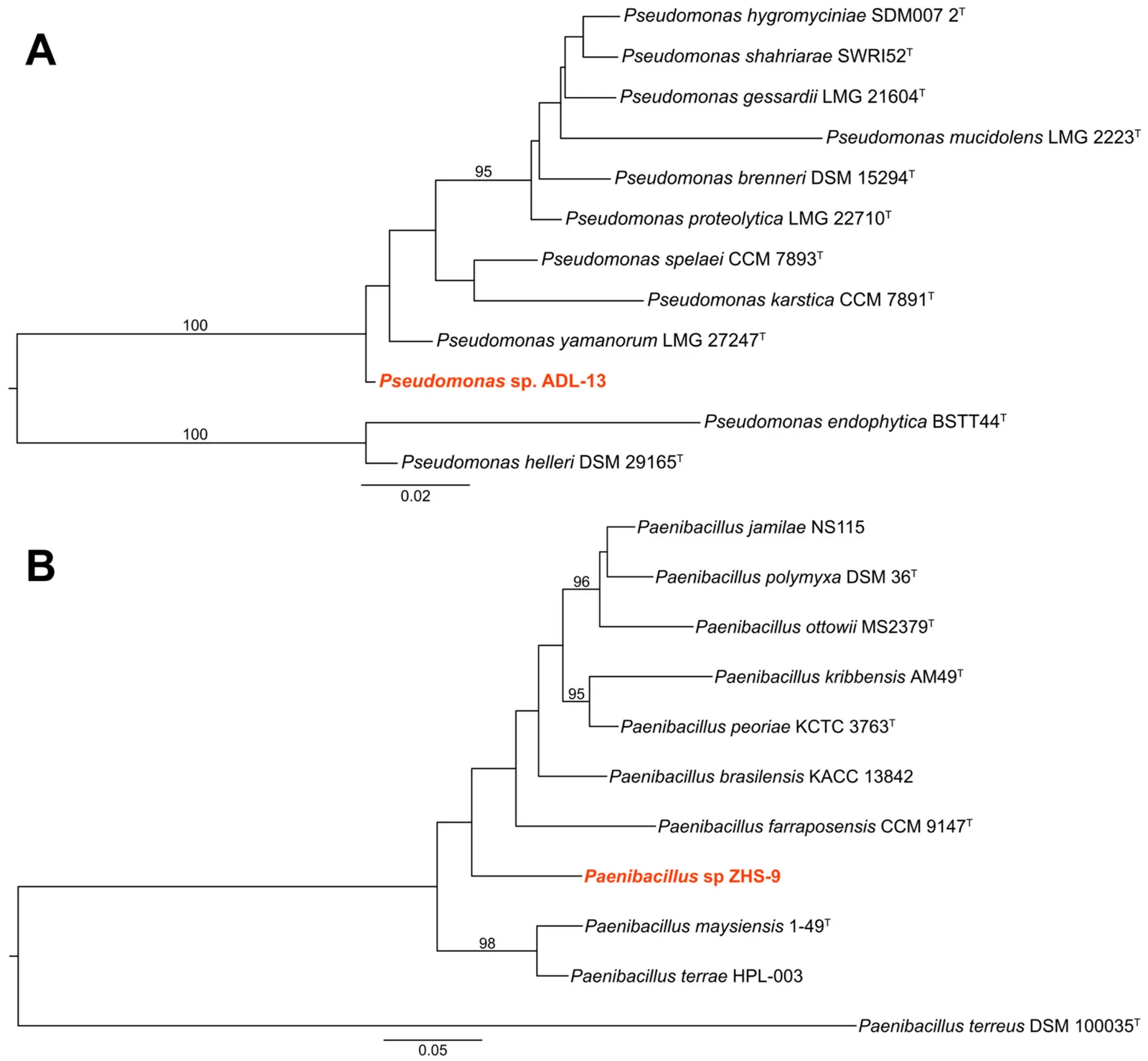
Maximum likelihood phylogram showing the phylogenetic position of *Pseudomonas* sp. ADL-13 (**A**) and *Paenibacillus* sp. ZHS-9 (**B**). Statistical support for the tree was calculated with UFBoot support. Only values higher than 95% are shown. Type strains are marked with superscript T.

The two most promising strains (ADL-13 and ZHS-9), selected based on their combined glyphosate tolerance (Section 3.2) and wheat promotion assays (Section 3.3), were subjected to the phylogenetic analyses (Figure 1).

Since *Pseudomonas* and *Paenibacillus* species are highly diverse genera comprising a wide range of species, 16S rRNA could not provide sufficient resolution for reliable species-level identification of ADL-13 and ZHS-9. Nevertheless, concatenated phylogenetic analysis of 16S rRNA with *gyrA* (for ZHS-9), *rpoB* and *rpoD* (for ADL-13) placed *Pseudomonas* sp. ADL-13 among environmental *Pseudomonas* species without close affiliation to recognized pathogenic taxa; whereas, *Paenibacillus* sp. ZHS-9 clustered within the *Paenibacillus polymyxa* species complex, which is predominantly composed of plant-associated and plant growth-promoting bacteria. For taxonomic precision, the strains are referred to as *Pseudomonas* sp. ADL-13 and *Paenibacillus* sp. ZHS-9, pending whole-genome sequencing. The *gyrA1* and *gyrA2* sequences of *Paenibacillus* sp. ZHS-9 and the *rpoB* and *rpoD* sequences of *Pseudomonas* sp. ADL-13 have been deposited in the NCBI GenBank database under accession numbers PZ771143, PZ771144, PZ771145, and PZ771146, respectively.

### 3.2. Glyphosate Tolerance

**Figure 2:**
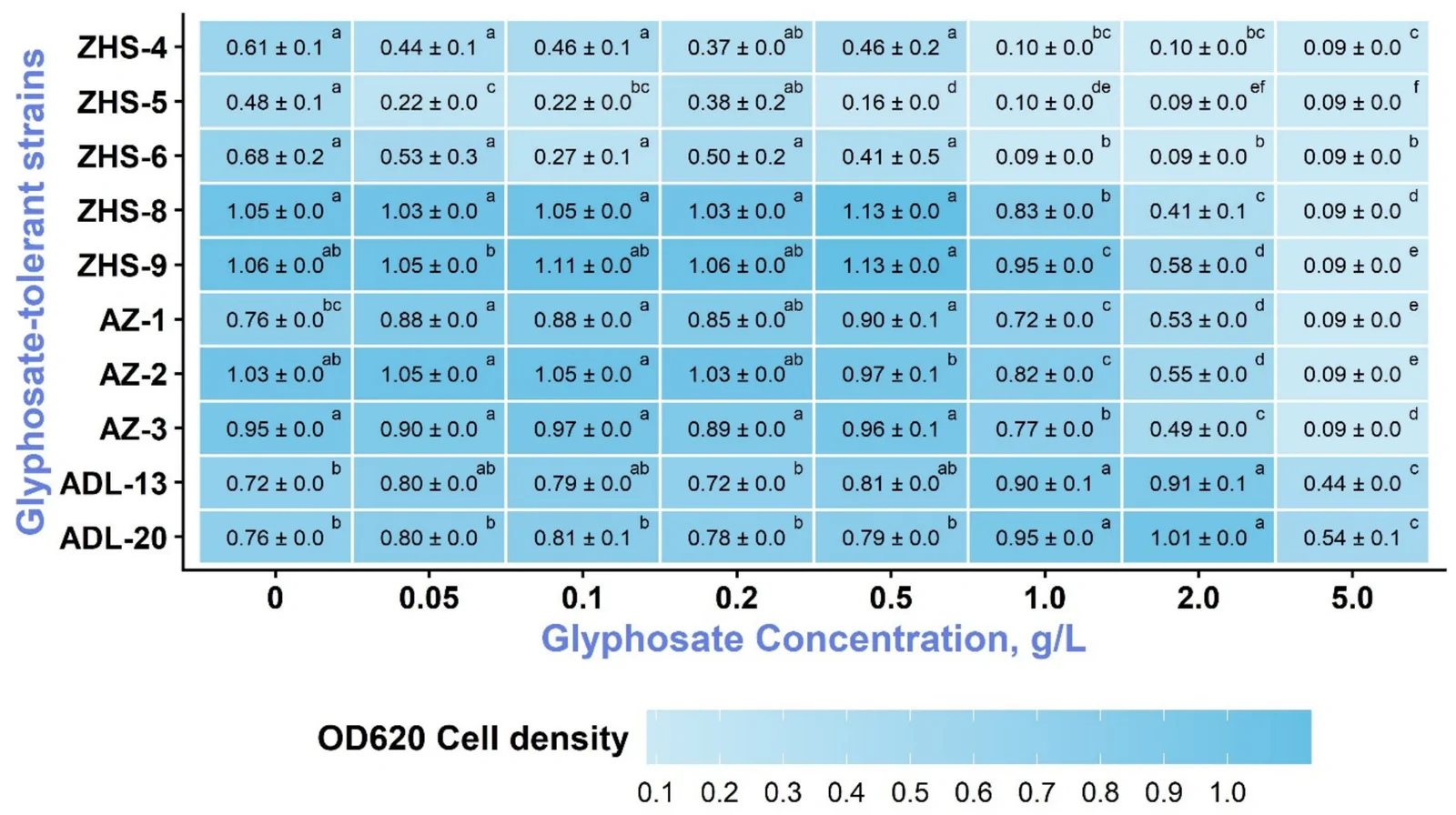
In vitro glyphosate tolerance of *Paenarthrobacter*, *Paenibacillus*, and *Pseudomonas* strains on liquid culture assays. Glyphosate concentrations are expressed as g/L glyphosate acid equivalent (a.e.) delivered through the commercial formulation. Heatmap values represent mean ± SD. Different letters (a–f) indicate significant differences across concentrations within each strain (*p* < 0.05, Tukey’s HSD).

The results of liquid culture assay covering a wide range of glyphosate concentrations at 0.05–5.0 g/L confirmed the glyphosate tolerance observed on solid medium (Section 3.1) for all steppe-derived isolates (Figure 2).

The majority of all isolates survived glyphosate concentrations up to 0.5 g/L without significant difference compared to the control without glyphosate (*p* > 0.05). Nevertheless, almost complete growth inhibition at 1.0 g/L glyphosate was observed in all three strains of *Paenarthrobacter*. In contrast, *Paenibacillus* strains demonstrated glyphosate tolerance up to 0.5 g/L without significant inhibition compared to the control (*p* > 0.05). Furthermore, although the growth of these strains was inhibited significantly at the higher 1.0 and 2.0 g/L glyphosate concentrations (*p* < 0.05), these isolates, especially ZHS-9, still demonstrated measurable cell density. Among all isolates, *Pseudomonas* strains ADL-13 and ADL-20 exhibited the highest glyphosate tolerance. Both strains displayed significantly greater growth at 1.0 and 2.0 g/L glyphosate (OD_620_ = 0.90–0.95 and 0.91–1.01 for ADL-13 and ADL-20, respectively), compared with the glyphosate-free controls (0.72 and 0.76, respectively; *p* < 0.05). Measurable growth was also maintained at the highest tested concentration of 5.0 g/L glyphosate (OD_620_ = 0.44 and 0.54 for ADL-13 and ADL-20, respectively), although significant inhibition was observed (*p* < 0.05).

Altogether, the results of both solid and liquid culture assays revealed species-specific and strain-dependent in vitro tolerance to glyphosate.

### 3.3. Wheat Seedling Bioassays

#### 3.3.1. The Effect of Cell-Free Bacterial Filtrates of ADL-13 and ZHS-9 on the Wheat Seedling Growth

The plant growth-promoting potential of bacterial metabolites was evaluated using cell-free culture filtrates (CCFs) in a wheat seedling bioassay (Table 2). Among the steppe-derived strains, only CCF from *Pseudomonas* sp. ADL-13 significantly increased fresh mass relative to both WC and the corresponding MC-I (*p* < 0.05). CCF from *Paenarthrobacter* sp. ZHS-4 significantly enhanced (*p* < 0.05) fresh mass and root length compared with MC-I (*p* < 0.05), while CCF from *Paenibacillus* sp. AZ-3 significantly increased fresh mass compared with WC (*p* < 0.05). However, none of these treatments differed significantly from both controls simultaneously. The results indicate that bacterial metabolites potentially presented in the CCF contributed positively to improved plant development.

Although *Paenarthrobacter* sp. ZHS-4 promoted fresh mass and root length significantly (*p* < 0.05), its growth was completely inhibited at glyphosate concentrations ≥1.0 g/L (*p* < 0.05, Figure 2), limiting its practical applicability under elevated glyphosate exposure. Conversely, *Paenibacillus* sp. ZHS-9 exhibited the greatest seedling length (15.68 mm) together with one of the highest total lengths (23.57 mm) and vigor indices (235.73). While these improvements were not statistically significant from WC or MC-II (*p* > 0.05), they demonstrated a consistent biological trend across multiple growth traits. In addition, ZHS-9 demonstrated the most sustained glyphosate tolerance up to 2.0 g/L among the *Paenibacillus* strains (Figure 2).

Therefore, strain selection was based on two complementary criteria considered jointly: (i) sustained glyphosate tolerance and (ii) positive responses across multiple plant growth traits in the CCF assay. *Pseudomonas* sp. ADL-13 exhibited one of the strongest glyphosate-tolerance profiles among the tested strains, while Paenibacillus sp. ZHS-9 showed the highest glyphosate tolerance among the tested Paenibacillus strains (Figure 2). Furthermore, both strains showed positive responses across multiple plant growth traits, particularly fresh mass, seedling length, and vigor index (Table 2). Therefore, ADL-13 and ZHS-9 were studied in all subsequent assays to characterize the plant growth-promoting traits and eco-physiological tolerance.

#### 3.3.2. The Effect of Bacterial Living Cultures of ADL-13 and ZHS-9 on the Wheat Seedling Growth

**Figure 3:**
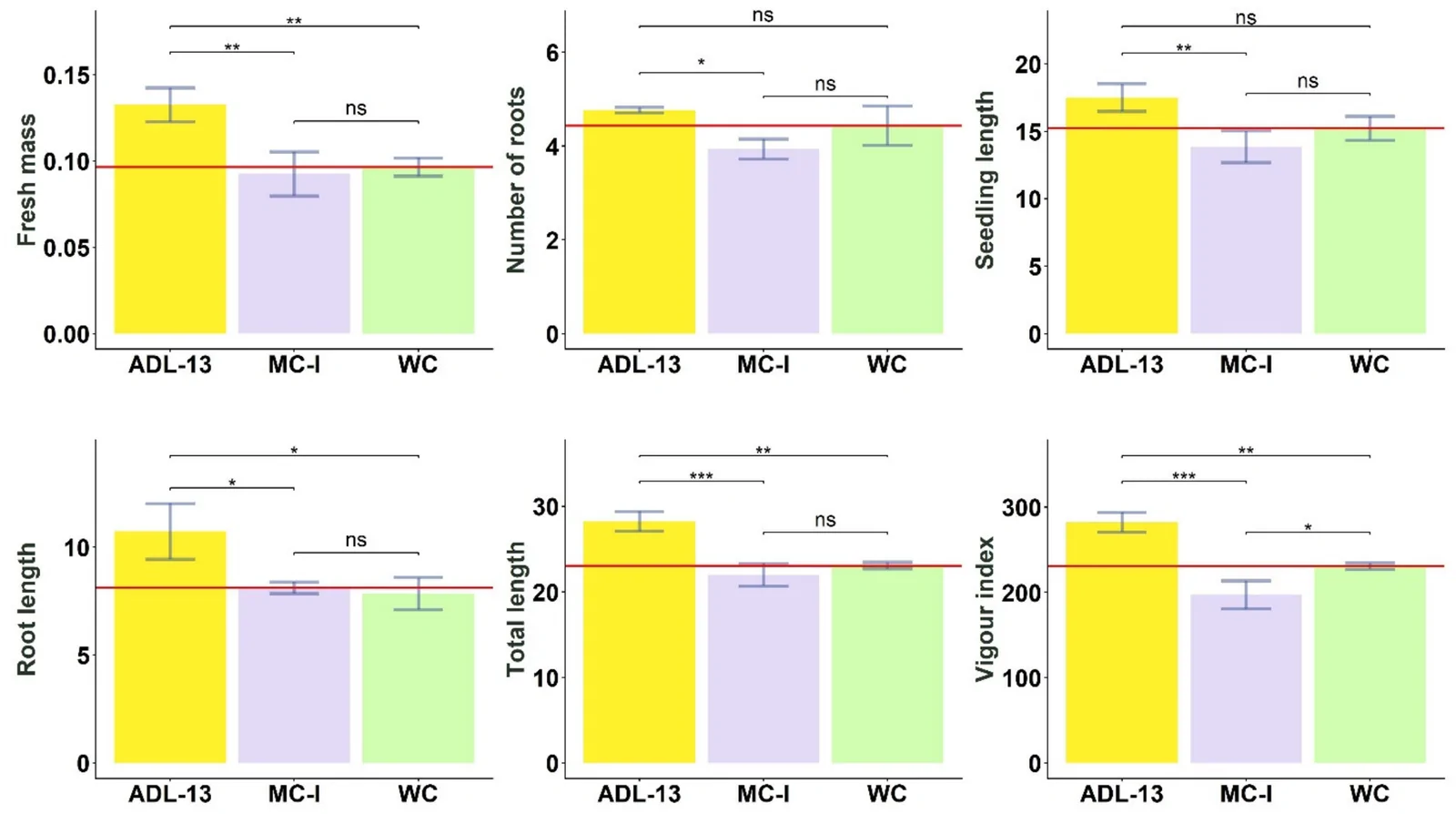
Effect of the living bacterial culture of *Pseudomonas* sp. strain ADL-13 on wheat seedling growth parameters compared to the distilled water control (WC) and MC-I control (MM amended with L-alanine and 0.1 g/L tryptophan). Statistical differences following Dunnett’s test are indicated as ns (not significant), *, **, and *** for *p* > 0.05, *p* < 0.05, *p* < 0.01, and *p* < 0.001, respectively. The red line represents the highest value between controls. Bars represent mean ± SD (*n* = 3 biological replicates; 10 seeds sown per pot).

The plant growth-promoting activity of the selected strains, *Pseudomonas* sp. ADL-13 and *Paenibacillus* sp. ZHS-9, was further evaluated using living bacterial culture in the identical wheat seedling bioassay employed for the CCF assays. The inoculation of seedlings with the living culture of ADL-13 resulted in improved plant development compared to both WC and MC-I controls (Figure 3). In particular, treatment with ADL-13 significantly improved most measured growth parameters, including fresh mass, root length, total length, and vigor index, relative to both controls. The number of roots and seedling length were significantly enhanced compared with MC-I but not with WC. These results indicate that inoculation with the living culture of strain ADL-13 positively influenced wheat seedling development (Figure 3).

Interestingly, while the CCF of ADL-13 significantly increased only fresh mass (*p* < 0.05, Section 3.3.1, Table 2), treatment with the LC enhanced fresh mass more strongly (*p* < 0.01) and, additionally, promoted root length (*p* < 0.05), total length (*p* < 0.01), and vigor index (*p* < 0.01).

**Figure 4:**
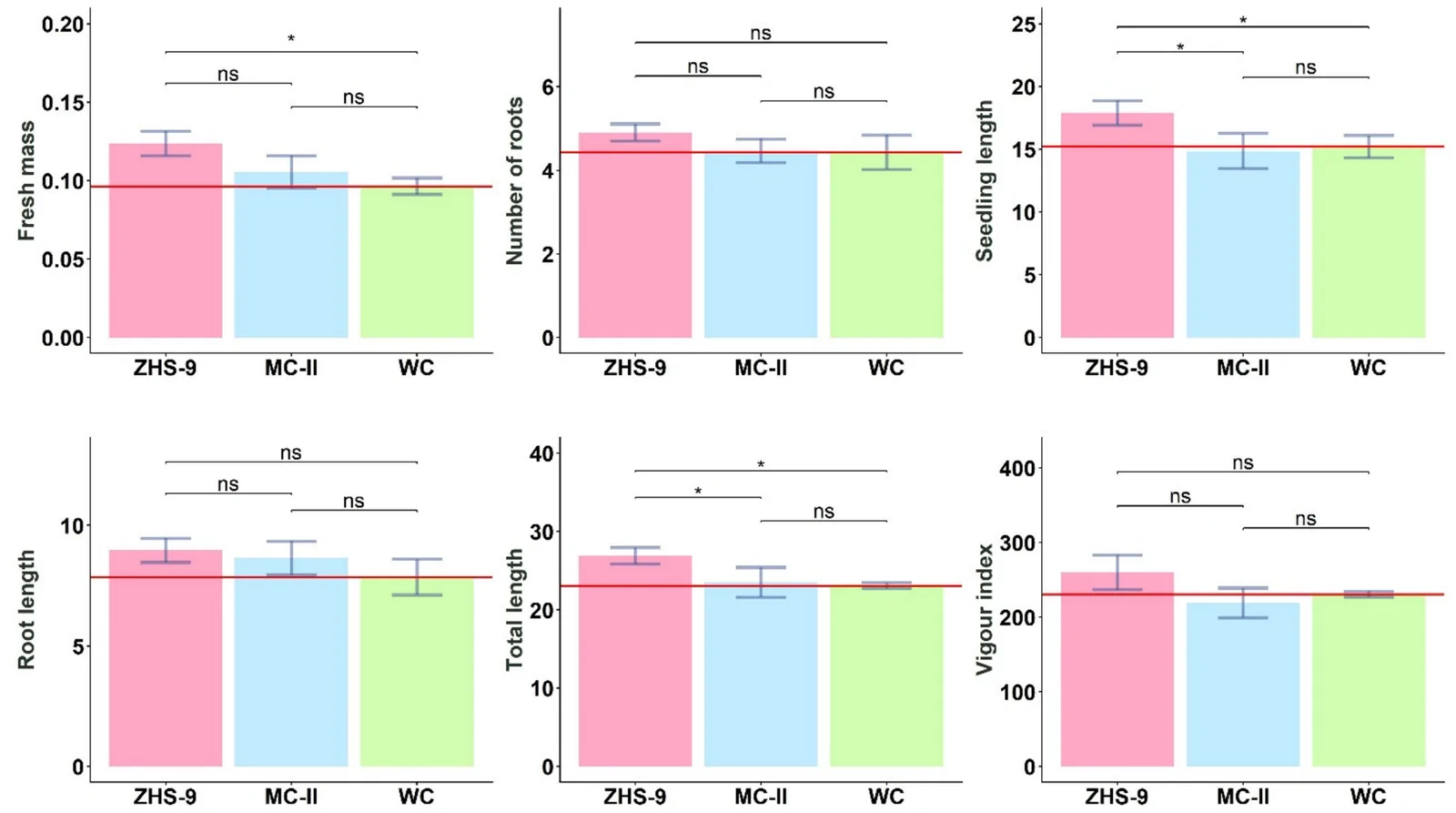
Effect of the living bacterial culture of *Paenibacillus* sp. strain ZHS-9 on wheat seedling growth parameters compared to the distilled water control (WC) and MC-II control (YEG supplied with 0.1 g/L tryptophan). Statistical differences following Dunnett’s test, are indicated as ns (not significant) and *, for *p* > 0.05, *p* < 0.05, respectively. The red line represents the highest value between controls. Bars represent mean ± SD (*n* = 3 biological replicates; 10 seeds sown per pot).

Treatments of wheat seedlings with living culture of *Paenibacillus* ZHS-9 strain also resulted in significant promotion of several wheat growth parameters (Figure 4). Application of the living culture of ZHS-9 significantly promoted seedling length and total length compared with both WC and MC-II controls (*p* < 0.05, Figure 4). Furthermore, inoculation with ZHS-9 significantly increased fresh mass compared with WC (*p* < 0.05), whereas the difference from MC-II narrowly missed statistical significance (*p* = 0.0579). Similarly, *p*-values of vigor index of the treatment with ZHS-9 showed a comparable trend, with *p*-values of 0.0531 and 0.1517 relative to MC-II and WC, respectively.

Interestingly, whereas the CCF of ZHS-9 showed a positive trend in seedling length and total length without reaching statistical significance (*p* > 0.05, Section 3.3.1, Table 2), treatment with the living cultures resulted in greater promotion of the same parameters, reaching statistical significance (*p* < 0.05).

These results indicate that CCF and inoculation with living cultures of *Pseudomonas* sp. strain ADL-13 and *Paenibacillus* sp. strain ZHS-9 were associated with wheat growth promotion. These observations suggest that living bacterial cells provide additional plant growth-promoting effects beyond extracellular metabolites alone.

### 3.4. PGP Characteristics

Both steppe-derived ADL-13 and ZHS-9 were subjected to a comprehensive examination of their plant growth-promoting profiles (Table 3).

*Pseudomonas* sp. ADL-13 demonstrated high tolerance to glyphosate, exhibiting no differences from the glyphosate-free control up to 0.5 g/L (*p* > 0.05), growth promotion at concentrations of 1.0–2.0 g/L (*p* < 0.01), and significant inhibition only at 5.0 g/L (*p* < 0.001). In wheat inoculation assays, this strain significantly enhanced fresh mass by 25.15–53.98% via its CCF (*p* < 0.05) compared with WC and MC-I, respectively. Under LC inoculation, ADL-13 consistently promoted multiple parameters, increasing fresh mass (37.33–43.14%), root length (36.67–32.13%), total length (22.31–28.42%), and the vigor index (22.31–43.13%) significantly (*p* < 0.05), relative to WC and MC-I, accordingly. Biochemically, ADL-13 solubilized dicalcium phosphate, tricalcium phosphate, and potassium. Siderophore production varied depending on the growth medium and carbon source (Table 3). In addition to producing ammonia in YEG and PW, ADL-13 also demonstrated other PGPB traits, including the OA production (Supplementary Figure S1) and biofilm formation (Supplementary Figure S2). Regarding synthesis of IAA-like indolic compounds and putative nitrogen-fixing ability (Supplementary Figure S3), ADL-13 did not exhibit these characteristics.

*Paenibacillus* sp. ZHS-9 displayed a glyphosate tolerance profile characterized by uninhibited growth promotion up to 0.5 g/L (*p* > 0.05) and significant inhibition only at 1.0–2.0 g/L (*p* < 0.01) yet showing measurable cell density. ZHS-9 inoculation showed a positive trend toward enhancing fresh mass, seedling length, and total length, via the CCF without statistical significance (*p* > 0.05). Nevertheless, the LC treatment consistently improved the same growth parameters as 28.25–17.33 for fresh mass, 17.64–20.49 for seedling length, and 16.45–14.35 for total length, reaching significant difference from both WC and MC-II (*p* < 0.05), with the exception of fresh mass compared with MC-II (*p* = 0.0579). Furthermore, ZHS-9 showed putative N_2_-fixing ability based on growth in N_2_-free Ashby’s medium (Supplementary Figure S3). Functionally, ZHS-9 solubilized dicalcium phosphate and potassium. This strain also synthesized substantial levels of IAA-like indolic compounds, yielding 20.97 µg/mL in YEG amended with L-tryptophan. Although ZHS-9 failed to produce ammonia in PW, in YEG it synthesized NH_3_ and 3.40 PSU. Additionally, the strain displayed localized clearing indicative of presumptive organic acid secretion, along with dense and well-developed biofilm (Supplementary Figures S1 and S2).

### 3.5. Eco-Physiological Profiling

#### 3.5.1. Tolerance to Abiotic Stress Factors and Optimal Temperature and pH Range

**Figure 5:**
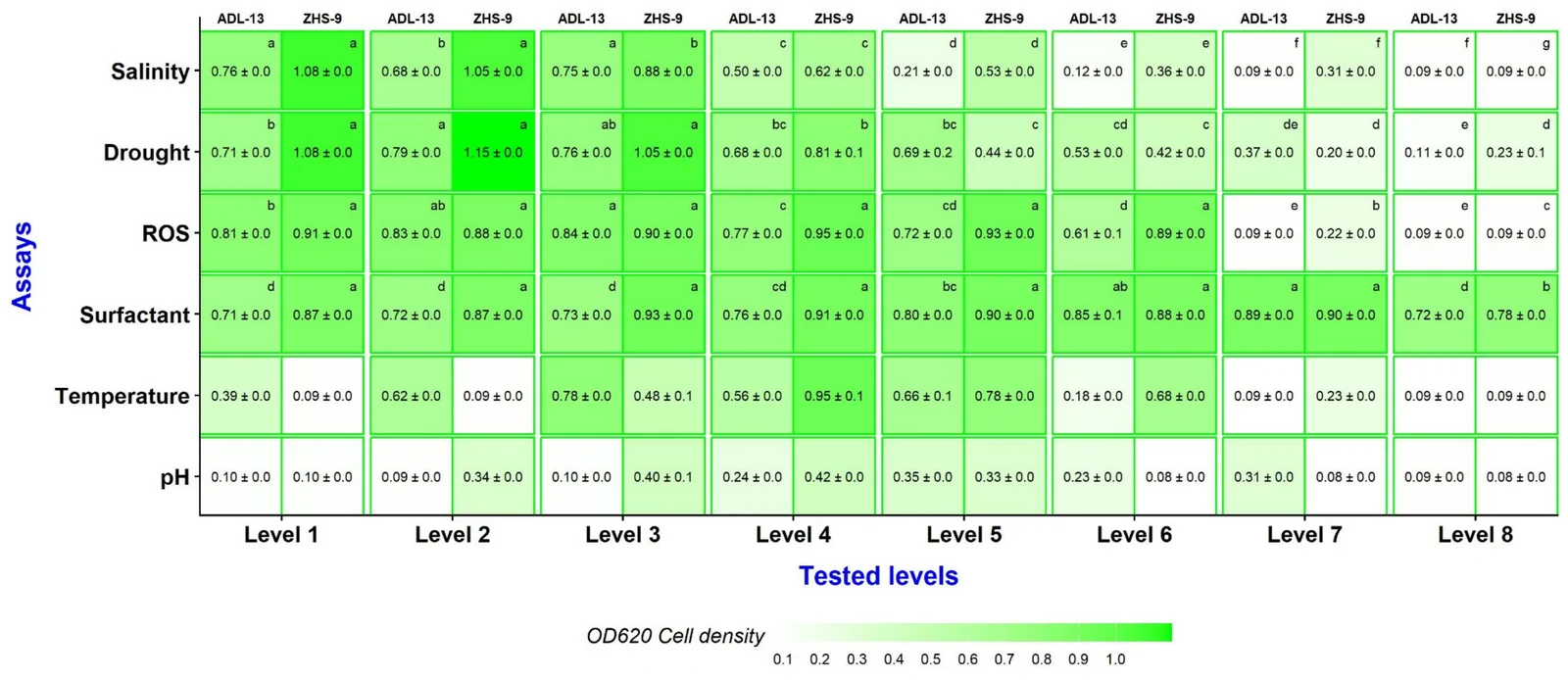
Eco-physiological profiling of *Pseudomonas* sp. ADL-13 and *Paenibacillus* sp. ZHS-9 strains. Split-cells display mean ± SD for strains ADL-13 (left half) and ZHS-9 (right half) across eight exposure levels. Significant differences following Tukey’s HSD test are indicated by different letters (a–g), indicating ANOVA-determined significant growth promotion or inhibition relative to untreated controls. The corresponding exposure levels (Level 1–8) are 0, 2, 5, 10, 15, 20, 25, and 50 g/L NaCl for salinity; 0, 25, 50, 100, 150, 200, 250, and 300 g/L PEG 6000 for drought; 0, 0.01, 0.05, 0.1, 0.2, 0.3, 0.5, and 1.0 mM H_2_O_2_ for reactive oxygen species (ROS) exposure; 0, 0.010, 0.025, 0.05, 0.1, 0.25, 0.5, and 1.0 % Tween 80 for surfactant; 10, 15, 20, 25, 30, 35, 40, and 45 °C for temperature; and 5.21, 6.38, 7.23, 7.85, 8.51, 9.21, 9.98, and 10.61 for pH, respectively.

The eco-physiological profiles of *Pseudomonas* sp. ADL-13 and *Paenibacillus* sp. ZHS-9 were evaluated under a range of abiotic stress conditions, including salinity, drought, oxidative stress (ROS), Tween, pH and temperature (Figure 5).

In this study, comprehensive eco-physiological profiles of the *Pseudomonas* sp. ADL-13 and *Paenibacillus* sp. ZHS-9 isolates were evaluated under different levels. Under salinity stress, strain ADL-13 exhibited substantial growth at low to moderate NaCl concentrations, with maximal growth observed at 5 g/L NaCl (Level 3). Growth declined significantly at 10 g/L NaCl (Level 4; *p* < 0.05), although measurable growth was still maintained. Complete inhibition occurred at NaCl concentrations of 20 g/L and above (Levels 6–8), indicating moderate halotolerance. Under drought stress induced by polyethylene glycol (PEG 6000), optimal growth occurred at 25–150 g/L PEG (Levels 2–5), whereas increasing osmotic stress (Levels 6–7) led to significantly reduced cell density (*p* < 0.05), with very limited growth at 300 g/L PEG (Level 8). The strain tolerated low to moderate concentrations of H_2_O_2_, maintaining measurable growth up to 0.3 mM (Level 6) despite significant inhibition (*p* < 0.05) yet stable growth with a sharp complete inhibition at 0.5–1.0 mM (Levels 7–8), demonstrating a measurable tolerance to oxidative stress. Surfactant tolerance assays showed that growth was significantly enhanced at 0.25 and 0.5% Tween 80 (Level 6–7; *p* < 0.05), whereas growth at all remaining concentrations did not differ significantly from the untreated control (*p* > 0.05), indicating strong tolerance to Tween exposure. ADL-13 exhibited optimal growth between 15 and 30 °C (Levels 2–5), whereas growth declined markedly at 35–45 °C (Levels 6–8), confirming its mesophilic growth characteristics. ADL-13 was capable of growth across a pH range of 7.85–9.98 (Levels 4–7), with optimal growth observed under slightly alkaline conditions.

*Paenibacillus* sp. ZHS-9 exhibited active growth under salinity stress up to 20 g/L NaCl (Levels 1–6). Although significant differences (*p* < 0.05) from NaCl-free control were observed at 5 g/L (Level 3), the strain maintained an OD_620_ of 0.36 or higher, with residual growth persisting up to 25 g/L (Level 7). Under PEG-induced drought stress, maximum cell density was achieved at 25 g/L (Level 2), with a gradual, significant decline (*p* < 0.05) occurring beyond 100 g/L (Level 4). ZHS-9 displayed robust oxidative stress tolerance, maintaining high cell densities across nearly the entire ROS gradient up to 0.3 mM (Levels 1–6) without significant differences (*p* > 0.05). Similarly, it adapted efficiently to the Tween gradient, maintaining stable statistically uninhibited cell density across all concentrations (Levels 1–7), except significant inhibition at Level 8. Compared with ADL-13, the thermal optimum for ZHS-9 shifted toward higher temperatures, showing growth between 20 °C and 40 °C (Levels 3–7) with the optimum as 25–35 °C (Levels 4–6). In terms of pH adaptability, ZHS-9 demonstrated wider tolerance toward neutral and moderately alkaline conditions, with optimal growth observed between pH 7.23 and 7.85 (Levels 3–4).

Overall, both ADL-13 and ZHS-9 strains demonstrated broad tolerance to multiple abiotic stress factors while maintaining substantial growth across a wide range of environmental conditions, highlighting their potential applicability in complex agricultural environments.

#### 3.5.2. Heat and Cold Shock Assays

**Figure 6:**
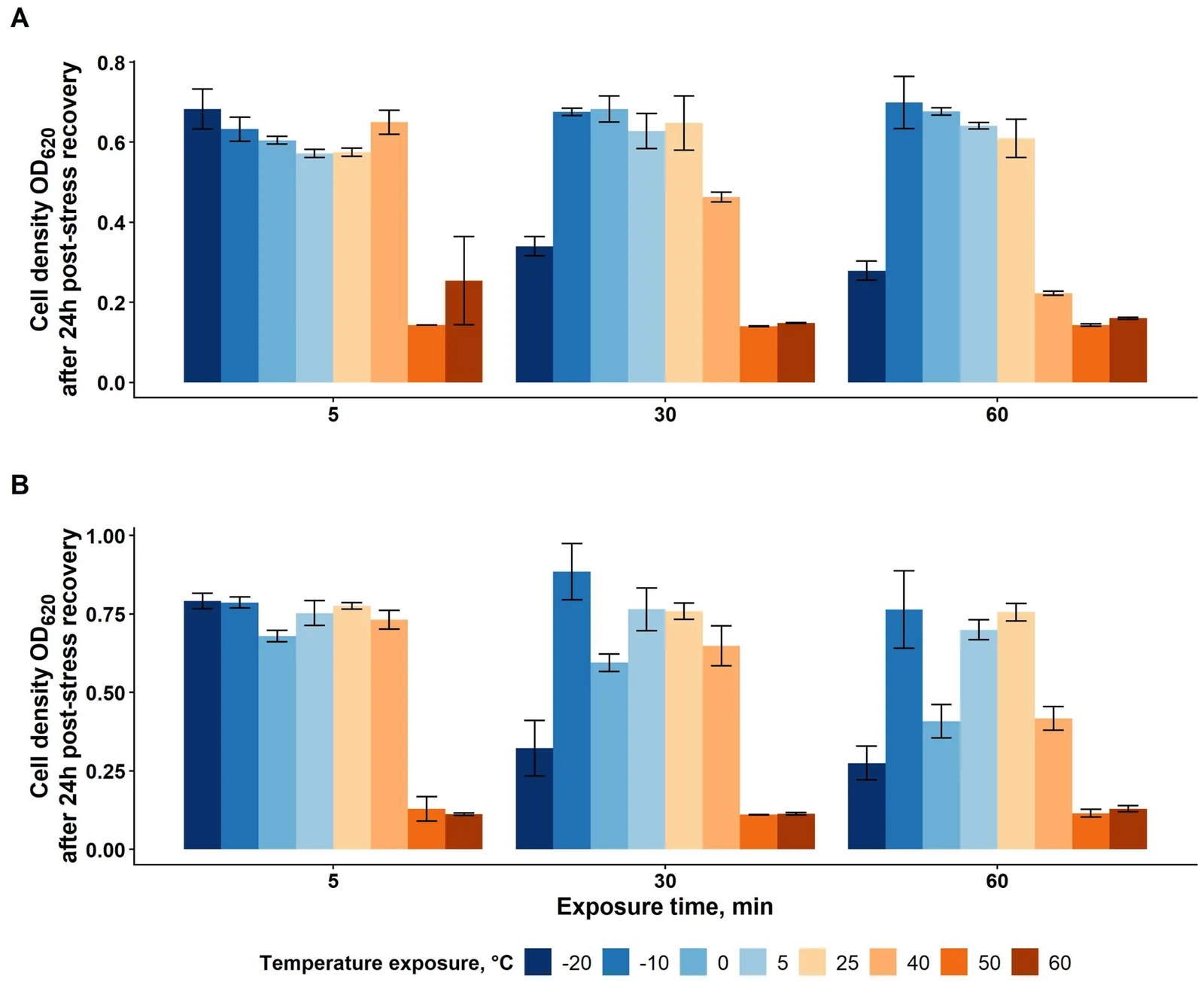
Evaluation of post-shock recovery following heat- and cold-shock treatments of glyphosate-tolerant *Pseudomonas* sp. ADL-13 (**A**) and *Paenibacillus* sp. ZHS-9 (**B**). Color gradient represents temperature exposure from blue (cold) to red (hot) levels.

The ability of *Pseudomonas* sp. ADL-13 and *Paenibacillus* sp. ZHS-9 to resume growth following abrupt temperature fluctuations was assessed using heat- and cold-shock assays with exposure durations of 5, 30, and 60 min (Figure 6).

The ADL-13 strain was capable of recovering across a broad range of shock temperature, with post-treatment growth observed following treatments ranging from −10 °C to 40 °C. After 60 min of exposure, growth following treatment at −20 °C was minimal, and a marked decline was observed following treatments within the 40–60 °C range, while the highest cell growth occurred following treatments between −10 °C and 25 °C. A similar pattern was observed after 30 min of exposure, with high growth maintained following treatments between −10 °C and 25 °C, and reduced growth following extreme temperature conditions. After 5 min of exposure, cell growth remained high across all tested temperatures, with the highest growth observed following treatments between −20 °C and 40 °C, whereas growth decreased following treatments at 50 and 60 °C.

The ZHS-9 strain demonstrated robust survival capabilities across a wide thermal spectrum, maintaining the ability to resume growth following treatments from −10 °C to 40 °C under varying exposure durations (Figure 6). Following 60 min of exposure, high post-treatment growth was observed following treatments within the −10 °C to 25 °C range. A low exposure (−20 °C) induced a sharp decline in post-treatment growth, with minimal growth recorded following treatments at the upper tested temperatures of 50 °C and 60 °C. A matching growth pattern was evident after 30 min of exposure, with the exception for 40 °C. Robust post-shock growth was sustained following treatments between −10 °C and 40 °C, while growth suppression occurred following treatments at −20 °C and 50–60 °C. Under short-term exposure (5 min), the strain retained measurable post-shock growth following most tested temperatures treatments. A remarkable drop in recovery was observed only when temperatures reached or exceeded 50 and 60 °C.

Overall, these results indicate that both ADL-13 and ZHS-9 strains retained post-shock growth across a range of transient temperature treatments.

#### 3.5.3. Qualitative Compatibility Assays

**Figure 7:**
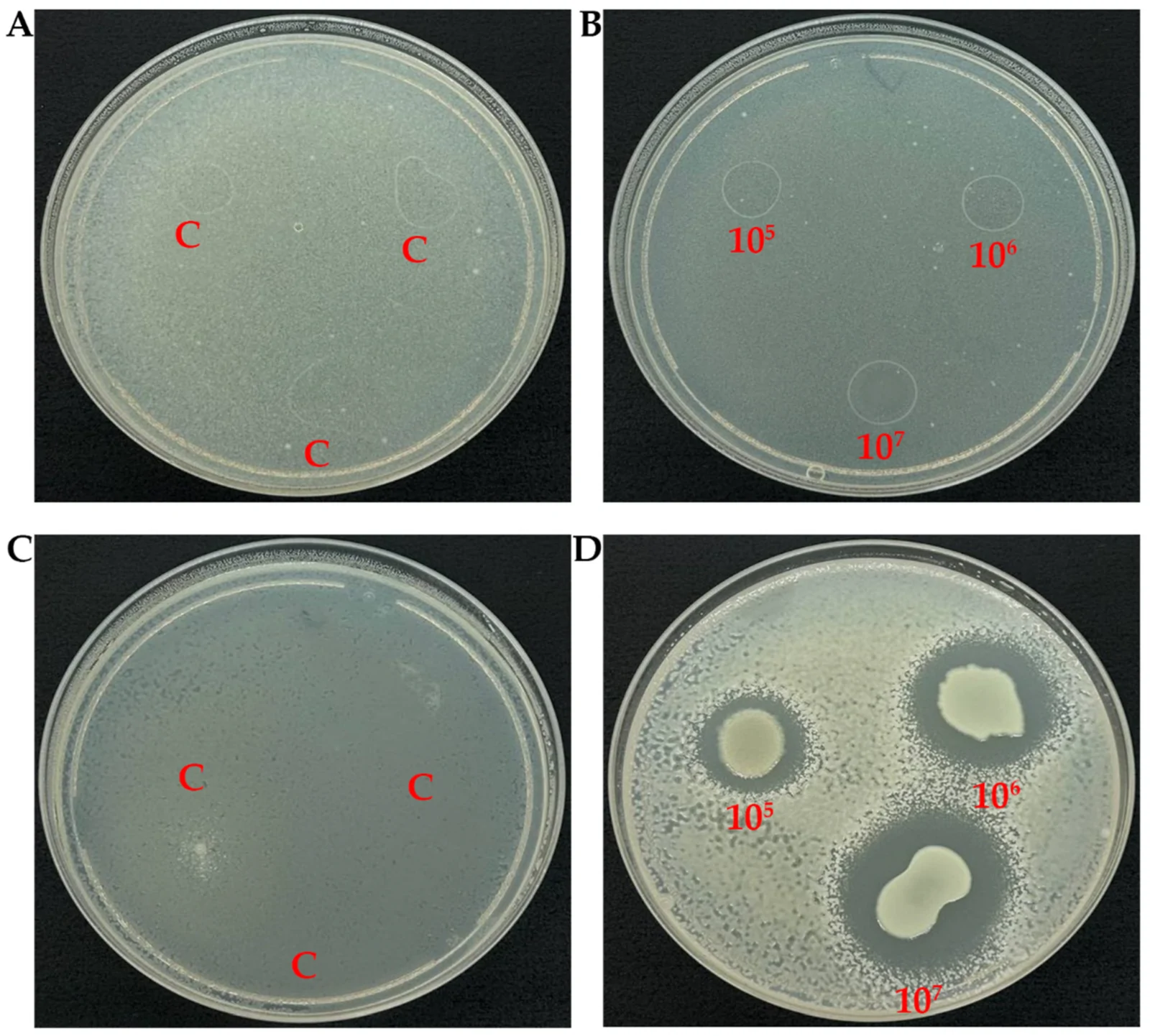
Compatibility assays demonstrating mutual interaction between *Pseudomonas* sp. ADL-13 and *Paenibacillus* sp. ZHS-9. *Pseudomonas* sp. ADL-13 inoculated solely as a control (**A**) and co-inoculated with *Paenibacillus* sp. ZHS-9 (**B**). *Paenibacillus* sp. ZHS-9 inoculated solely as a control (**C**) and co-inoculated with *Pseudomonas* sp. ADL-13 (**D**). The label “C” indicates the control treatment containing sterile 0.9% NaCl used for preparing all bacterial suspensions. Labels “10^5^”, “10^6^”, and “10^7^” indicate different inoculum concentrations expressed as CFU/mL.

Since ADL-13 and ZHS-9 individually promoted wheat growth, their potential combined effects as a microbial consortium were considered. Before evaluating such consortium, the compatibility assay was performed to assess mutual interactions between the two strains (Figure 7).

The compatibility testing revealed no inhibitory effect of ZHS-9 against ADL-13 (Figure 7B). However, antagonistic activity of ADL-13 on ZHS-9 was observed (Figure 7D), consequently preventing their use as compatible consortium. Nevertheless, both strains remain promising candidates as individual biofertilizers because each independently promoted wheat seedling growth (Section 3.3) and exhibited multiple PGP mechanisms (Section 3.4). This test also demonstrates the importance of co-culture compatibility testing to evaluate mutual interactions between strains, prior to the assembly of their consortia.

#### 3.5.4. SEM/TEM

**Figure 8:**
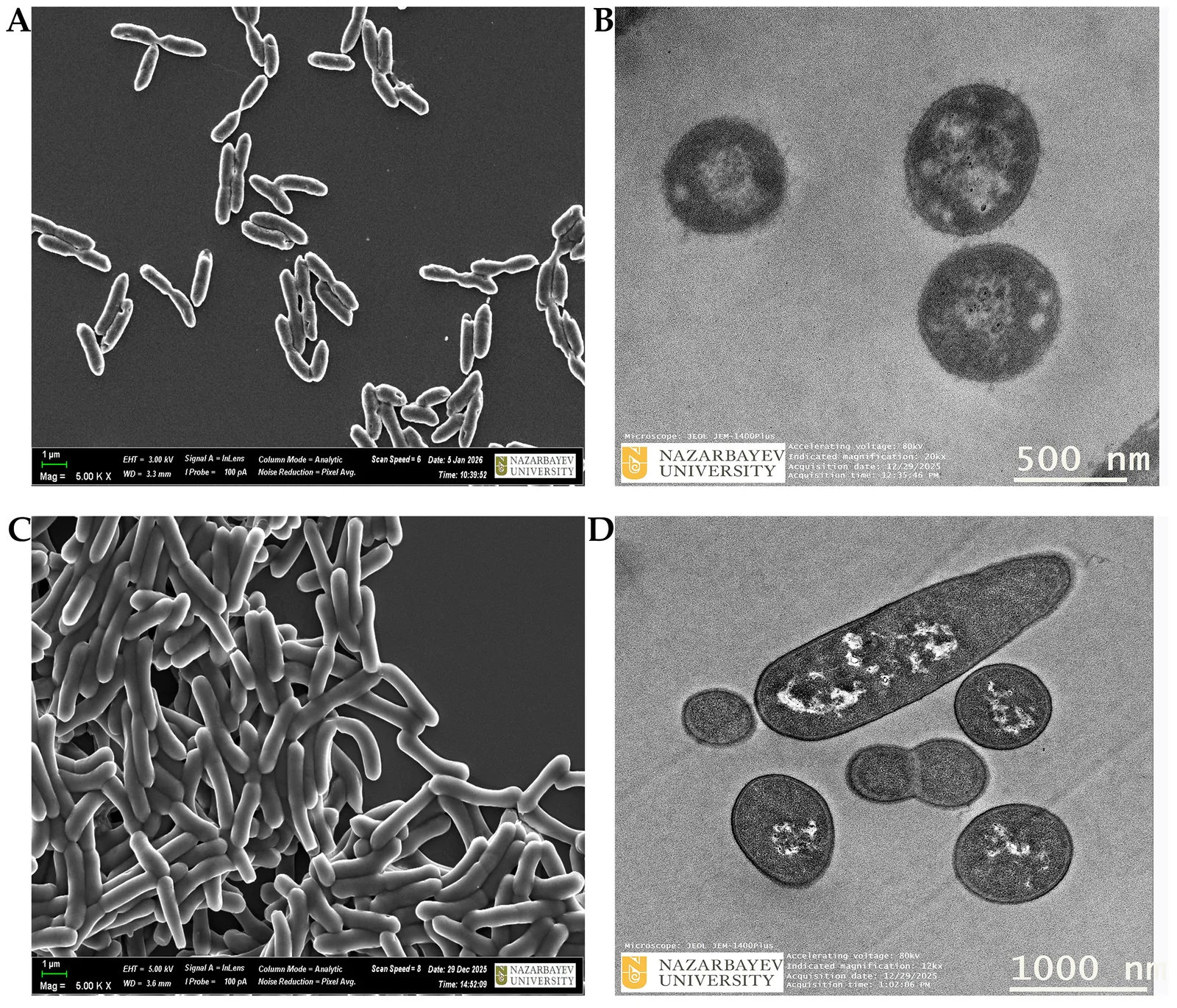
SEM and TEM images illustrating the cellular morphology of *Pseudomonas* sp. ADL-13 ((**A**) and (**B**), respectively) and *Paenibacillus* sp. ZHS-9 ((**C**) and (**D**), respectively).

The cellular morphology of ADL-13 and ZHS-9 was characterized using scanning electron microscopy (SEM) and transmission electron microscopy (TEM) (Figure 8).

Ultrastructural examination via electron microscopy confirmed the typical morphological characteristics of the isolates (Figure 8). SEM images revealed that the cells of both *Pseudomonas* sp. ADL-13 (Figure 8A) and *Paenibacillus* sp. ZHS-9 (Figure 8C) are rod-shaped, displaying smooth outer surfaces and intact cell walls. Complementing these surface observations, TEM images provided visualization of the general internal cellular architecture of the strains (Figure 8B,D). The cytoplasm appeared relatively uniform, with general internal structural features. All original images are included in the Supplementary Figures S4–S7.

Collectively, these analyses offer a comprehensive characterization of the cellular and subcellular morphology of the ADL-13 and ZHS-9 isolates, providing a foundational baseline for ultrastructural characteristics, supporting their phenotypic and taxonomic characterization, and complementing the physiological and biochemical characterization presented above.

## 4. Discussion

### 4.1. Glyphosate Tolerance of Steppe-Derived Bacterial Strains

Crop productivity is increasingly threatened by a combination of abiotic stresses, including drought, salinity, temperature extremes, nutrient deficiencies, and heavy metal toxicity, many of which are exacerbated by ongoing climate change [14]. Among these stressors, glyphosate is one of the most widely used herbicides worldwide [52] and represents an additional anthropogenic stressor in agricultural ecosystems. However, its extensive application has raised environmental concerns, as excessive use may negatively affect ecosystems, reduce biodiversity, and impair soil health [53]. Therefore, the ability of PGPB to tolerate glyphosate is an important ecological trait that may contribute to their survival and functionality in agricultural soils. Considering that agricultural soils are often exposed to glyphosate residues, assessing the herbicide tolerance of candidate PGPB is essential to ensure their survival and effectiveness under field conditions [22].

In the present study, to enrich for glyphosate-tolerant bacterial strains, soil samples were collected from agricultural fields with a documented history of repeated glyphosate exposure. This prior environmental exposure may have contributed to selection for indigenous microbial populations capable of enduring herbicide stress. Additionally, a two-step screening strategy was employed, consisting of an initial selection on PMM supplemented with glyphosate as a selective barrier, followed by quantitative assessment of bacterial growth in liquid YEG containing a broad range of glyphosate concentrations. Consequently, all ten isolates representing three genera (*Paenarthrobacter*, *Paenibacillus*, and *Pseudomonas*) tolerated glyphosate concentrations ranging from 0.5 to 2.0 g/L, depending on the species and individual strain (Figure 2). Among these isolates, both selected strains *Pseudomonas* sp. ADL-13 and *Paenibacillus* sp. ZHS-9 exhibited a high level of herbicide resilience, showing no significant growth inhibition at glyphosate concentrations up to 0.5 g/L (*p* > 0.05). ADL-13 demonstrated significant growth stimulation at 1.0 and 2.0 g/L glyphosate (*p* < 0.01), whereas ZHS-9 maintained measurable, although significantly inhibited, growth under the same concentrations (*p* < 0.01). These findings suggest a considerable level of glyphosate tolerance, within the range of concentrations previously used for the isolation and screening of glyphosate-tolerant bacteria [22,54]. Nevertheless, the concentrations tested in this study were selected for laboratory isolation and tolerance screening and should not be interpreted as directly representative of glyphosate concentrations occurring in agricultural soils. The observed in vitro glyphosate tolerance of the isolates indicates their potential for further evaluation under glyphosate exposure in agricultural environments. In addition, these strains represent glyphosate-tolerant bacterial strains derived from agricultural steppe environments. Our 2021 study characterized glyphosate-tolerant *Ensifer adhaerens* SZMC 25856 and *Pseudomonas resinovorans* SZMC 25875 from agricultural soil in northern Serbia and evaluated their PGP properties, effects on tomato growth, and stress-related properties [23]. In our previous study, *Paenibacillus* sp. JSM-10 also demonstrated glyphosate tolerance coupled with buckwheat growth enhancement and antagonistic activity against a range of pathogenic microorganisms [22]. In the present study, ADL-13 and ZHS-9 are previously uncharacterized isolates, and their characterization was performed in a different experimental system using wheat, separate assessment of cell-free culture filtrates and living cells, baseline PGP profiles, a distinct abiotic-stress panel, and direct inter-strain compatibility testing. Three strains-CR14, CR18 and CR22 isolated from a geographically distinct region of Kazakhstan also demonstrated plant growth-promoting and biocontrol potential [55]. Collectively, these findings indicate that agricultural steppe soils remain an underexplored yet promising source of novel PGPB with diverse plant-beneficial characteristics.

### 4.2. Growth-Promoting Potential of ADL-13 and ZHS-9

PGPB serve as indispensable drivers of biogeochemical processes, actively regulating soil fertility, accelerating nutrient cycling, and fostering vegetative development [56]. Bacterial species belonging to the genera *Paenibacillus* and *Pseudomonas* are among the most extensively studied PGPB due to their well-documented beneficial effects on plant growth [57].

The present study demonstrated that the newly isolated, glyphosate-tolerant strains *Pseudomonas* sp. ADL-13 and *Paenibacillus* sp. ZHS-9 significantly enhanced the early establishment of wheat seedlings (*T. aestivum* L. cv. “Aqmola-2”). The plant growth-promoting potential of bacterial metabolites was evaluated using both CCF and LC to evaluate the effect of extracellular metabolites and living cells, respectively (Section 3.3.1 and Section 3.3.2). Both isolates exerted pronounced growth-promoting effects, resulting in substantial increases in fresh mass, seedling length, lateral root development, and overall seed vigor. In CCF treatment, the comparatively lower values observed in MC-I and MC-II indicate that wheat promotion in bacterial treatment were associated with bacterial extracellular metabolites rather than with the medium components alone (Table 3). However, as ADL-13 and ZHS-9 CCFs were obtained using different growth media, direct quantitative comparison of their CCF effects should be interpreted with caution, despite the use of corresponding medium controls. In the LC treatment, even greater growth-promoting effects were observed, suggesting that the presence of living bacterial cells enhanced plant growth (Figure 3 and Figure 4).

These findings are consistent with previous reports describing members of the genera *Pseudomonas* and *Paenibacillus* as effective PGPB capable of improving crop performance under diverse environmental conditions [58,59]. Table 4 provides an overview of plant growth responses, including biomass accumulation, root development, seedling growth, and vigor-related parameters, described for ADL-13, ZHS-9, and previously reported *Paenibacillus* and *Pseudomonas* strains evaluated in wheat and barley.

Assessment of early seedling growth parameters, including root and shoot elongation and biomass accumulation, is widely used for screening and selecting bacterial strains with plant growth-promoting potential [69]. The significant enhancement of such growth metrics observed in this study indicates the plant growth-promoting activity of ADL-13 and ZHS-9 under controlled bioassay conditions. Consequently, these findings highlight the notable plant growth-promoting potential of both steppe-derived isolates during early plant establishment and support their further evaluation under field and soil conditions for sustainable agriculture.

### 4.3. Plant Growth-Promoting Profiling

It is well established that PGPB enhance plant growth through a combination of direct physiological stimulation and indirect protective mechanisms [70]. These beneficial effects are attributed to various traits, including iIAA production, phosphate solubilization, siderophore secretion, nitrogen fixation, and ACC deaminase activity, which collectively improve nutrient availability, root development, and plant stress tolerance [71].

In the present study, glyphosate exposure was used primarily as a selection criterion during bacterial isolation and subsequent tolerance screening, whereas PGP traits were characterized in vitro under standardized conditions without additional glyphosate supplementation. This experimental workflow was intentionally designed to first select and assess strains for tolerance to the glyphosate-based herbicide formulation and subsequently characterize their baseline PGP profiles independently of potential herbicide-induced physiological effects. Establishing an initial baseline of their PGP characteristics is therefore important for subsequent evaluation of more complex chemical–microbe interactions in these steppe-derived strains. Consequently, the PGP traits reported here should be interpreted as baseline functional characteristics of the selected strains rather than as evidence that these traits remain unchanged under glyphosate stress. This represents a limitation of the present study. Future studies should therefore assess PGP trait expression and plant growth promotion under glyphosate exposure, as well as the optimal interval between herbicide treatment and PGPB application and the number and timing of subsequent bacterial applications.

*IAA-like indolic compounds.* In the present study, no IAA-like indolic compounds were detected in the bacterial supernatant of *Pseudomonas* sp. ADL-13 isolate, whereas *Paenibacillus* sp. ZHS-9 isolate produced 20.97 µg/mL in YEG amended with 0.1 g/L tryptophan (Table 3). The detected level of IAA-like indolic compounds falls within the range of values reported using related colorimetric assays; however, direct numerical comparisons among studies are limited by differences in media, tryptophan supplementation, incubation conditions, and analytical specificity. For instance, *Bacillus pretiosus* (C1) has been reported to produce 5.61 µg/mL of IAA [72], while *Pseudomonas fluorescens* CHA0 exhibited a significantly higher production of 88.37 µg/mL [73].

*Phosphorus solubilization*. As phosphorus (P) is an essential macronutrient for plant growth and development, phosphate solubilization is considered a critical PGP mechanism because it increases phosphorus availability for plants and may improve crop yield [74,75]. In this study, the isolates demonstrated different capacities to solubilize phosphate sources (Table 3). ADL-13 exhibited efficiency, with PSI of 1.9 for CaHPO_4_ and 1.13 for Ca_3_ (PO4)_2_. In contrast, the ZHS-9 showed moderate PSI of 1.28 for CaHPO_4_ but failed to produce a visible halo on Ca_3_ (PO4)_2_. These differences may reflect strain-specific phosphate-solubilization mechanisms, including production of organic acids. Moreover, tricalcium phosphate is inherently more stable and less soluble than dicalcium phosphate, making it more difficult to solubilize [76]. The importance of phosphate-solubilizing bacteria for plant nutrition has also been demonstrated in previous studies. For example, inoculation with *Pseudomonas* sp. WS32 increased phosphorus accumulation in wheat plants by 49.42% compared with uninoculated controls [77]. Collectively, these findings suggest that both isolates possess promising phosphate-solubilizing potential, which may contribute to improved phosphorus availability and enhanced plant growth.

*Potassium solubilization* is another important PGP trait because potassium (K) is an essential macronutrient required for plant growth and several physiological processes, including osmotic regulation, stomatal movement, and protein synthesis [78]. In our strains, ADL-13 and ZHS-9 demonstrated KSI of 1.11 and 1.20, respectively, further supporting its multifunctional plant growth-promoting potential (Table 3). The ability to solubilize both potassium and phosphorus suggests that ADL-13 and ZHS-9 may improve plant nutrition through nutrient acquisition mechanisms.

*Siderophores production.* Iron (Fe) is a vital micronutrient required for plant development, and its importance is largely attributed to its unique chemical characteristics that enable participation in redox reactions. These properties allow Fe to play essential roles in key biological processes, including photosynthesis, respiration, and chlorophyll biosynthesis [79,80]. In our study, the siderophore production was highly dependent on the carbon source and varied from 13.35 PSU in complex YEG medium to 33.93 in iron-depleted SMM supplied with succinic acid as a sole carbon source with the maximum of 63.05 PSU in the same medium amended with glucose (Table 3). *Paenibacillus* sp. ZHS-9 failed to grow in SMM supplied with either succinic acid or glucose yet exhibited a modest level of 3.40 PSU in the complex YEG medium. The previous studies reported siderophore production ranging from 19.69 to 31.02% SU for strain *Pseudomonas putida* Pt12 and 13.46 μg/mL for *Paenibacillus triticisoli* BJ-18 under Fe-deficient conditions [81,82].

*Putative nitrogen-fixing ability and ammonia production.* Nitrogen is also a vital nutrient for plant development, forming a fundamental component of amino acids, proteins, chlorophyll, and nucleic acids [83]. In the present study, the continuous growth over one-week incubation indicated putative nitrogen-fixing ability of *Paenibacillus* sp. ZHS-9 while no growth was observed in *Pseudomonas* sp. ADL-13 (Supplementary Figure S3). These findings are in accordance with the currently available information reporting 20 species with reported nitrogen fixation potential out of 150 known *Paenibacillus* species [84]. Weselowski et al. [85] assessed nitrogen-fixing ability of the *P. polymyxa* CR1 strain also by its growth in nitrogen-free medium, further confirmed with the presence of the *nif* gene cluster. In contrast, the present study provides only phenotypic evidence based on growth in nitrogen-free medium, and the nitrogen-fixing ability of ZHS-9 therefore remains putative and requires molecular and/or functional confirmation.

In addition to nitrogen fixation, ammonia production is a valuable nitrogen source. Intriguingly, in the present study, ammonia production by the two strains exhibited strict substrate dependency. While only *Pseudomonas* sp. ADL-13 demonstrated 26.43 µM ammonia accumulation in standard peptone water, both ZHS-9 and ADL-13 successfully secrete 8.31 and 11.53 µM ammonia when cultivated in YEG medium, respectively. Because the two media were sampled at different incubation times, ammonia concentrations should be interpreted within each medium and should not be used for direct quantitative comparison between YEG and peptone water. This finding suggests that ammonia production may have been influenced by the composition of the growth medium, although the underlying mechanism remains to be determined.

*OA secretion.* Calcite solubilization is considered a valuable geochemical process regulated by soil microorganisms producing organic acids, mainly including citric, gluconic, and acetic acids [41,86]. ADL-13 formed prominent, clearly visible halo zones on Ashby’s medium supplied with CaCO_3_. In contrast, ZHS-9 produced a fainter and more localized clearing that was consistently detectable by direct visual inspection but was less clearly defined in the photograph. Therefore, the ZHS-9 phenotype is considered here as exhibiting presumptive OA secretion, which warrants further quantitative confirmation. *Azotobacter salinestris* YRNF3 was reported to have calcite-solubilizing potential to improve soil properties and plant growth in calcareous soils [41]. Similarly, our results demonstrate that both strains are capable of mineral solubilization under the tested laboratory conditions, highlighting their relevance for further investigation of their role in mineral mobilization in calcareous agricultural soils.

*Biofilm formation.* The biofilm-forming ability was evaluated using the crystal violet microtiter plate assay. ADL-13 and ZHS-9 formed biofilms under static conditions, with average optical densities of 3.25 ± 0.57 (a) and 1.30 ± 0.35 (b), respectively, which were significantly higher than the negative control (0.25 ± 0.03; (c)). Biofilm formation provides bacteria with numerous survival advantages, protecting bacterial cells from external stresses, thereby enhancing their survival and persistence [56]. In plant-associated bacteria, biofilm formation is considered an important trait that facilitates root surface attachment and stable colonization of the rhizosphere [87]. Therefore, the ability of ADL-13 and ZHS-9 to form stable biofilms contributes to the plant growth-promoting activity.

### 4.4. Eco-Physiological Adaptations and Environmental Resilience

To comprehensively evaluate the environmental profiles of the isolates, we assessed not only commonly investigated parameters such as pH, temperature, salinity, and osmotic stress tolerance but also less frequently examined traits, including oxidative stress tolerance and surfactant resistance, which provide additional insight into physiological robustness (Figure 5).

*Salinity tolerance*. Soil salinization is recognized as a critical environmental problem that significantly alters the structure, processes, and functioning of ecosystems worldwide [88]. Since agricultural soils are often exposed to multiple concurrent stresses, including herbicide residues and salinity, the assessment of PGPB performance under salt stress is crucial for their potential application in field conditions. Our results demonstrated that both isolates maintained growth at 15 g/L NaCl, although cell density was significantly reduced compared to the untreated controls (*p* < 0.05). Previous studies have reported comparable salt tolerance boundaries in PGPB; for instance, *Paenibacillus polymyxa* strains frequently tolerate up to 2% NaCl, while certain *Pseudomonas* spp. exhibit tolerance ranges from 6% to 8% under diverse growth conditions [89,90].

*Drought tolerance*. Besides salinity tolerance, both isolates exhibited considerable resilience to osmotic stresses. No significant growth inhibition was observed for ADL-13 at 50–150 g/L and for ZHS-9 up to 50 g/L PEG-6000 (*p* > 0.05), indicating strain-specific tolerance to drought-induced osmotic stress. In addition, both strains remained viable across a broad range of temperatures and pH values (pH 7.85–9.98 for ADL-13 and pH 6.38–8.51 for ZHS-9). Similar findings have been reported for other PGPB. For example, *Bacillus* sp. D13, *Pantoea agglomerans* Pa, *Pseudomonas knackmussi* MR6, and *Providencia vermicola* ME1 maintained plant growth-promoting activities under osmotic stress induced by 10–30% PEG-6000 [91]. These results, highlighting their strong eco-physiological adaptability, relevant for steppe microorganisms experiencing rapid environmental fluctuations.

*Oxidative stress tolerance*. Although hydrogen peroxide is known to degrade relatively rapidly in complex growth media due to catalytic activity and interactions with medium components, its initial presence creates a transient but physiologically relevant oxidative stress. In this study, we applied defined concentrations of H_2_O_2_ (0.01–1.0 mM) to freshly inoculated cultures in YEG medium within 96-well microplates and monitored bacterial growth over 48 h. As H_2_O_2_ may degrade during the incubation period, subsequent bacterial recovery and outgrowth may reflect the strain’s tolerance mechanisms, potentially involving ROS detoxification, DNA repair, and membrane integrity preservation. Hence, measurable OD_620_ after incubation serves as an integrative indicator of bacterial growth and fitness under the applied oxidative stress conditions. *Paenibacillus* sp. ZHS-9 survived up to 0.3 mM H_2_O_2_ without significant inhibition (*p* > 0.05). *Pseudomonas* sp. ADL-13 grew without significant inhibition up to 0.05 mM H_2_O_2_ (*p* > 0.05) while significantly inhibited (*p* < 0.05) but with measurable cell density recorded up to 0.3 mM.

*Surfactant tolerance*. The surfactant Tween 80 is widely used in commercial microbial formulation to extend shelf-life [92]; however, its effect on planktonic growth was reported as species-specific [45]. Therefore, the robust tolerance of both studied glyphosate-tolerant strains to the presence of Tween 80 up to 0.5% without significant inhibition (*p* > 0.05) suggests their suitability for the potential formulation with polysorbate surfactants to extend commercial shelf-life.

*Thermal and pH adaptability.* Both isolates tolerated broad ranges of pH and temperature, demonstrating considerable physiological adaptability. Such flexibility is advantageous for agricultural applications, as soil pH and temperature vary substantially under field conditions, particularly in steppe environments characterized by pronounced seasonal fluctuations. In addition to sustained growth across different temperatures, both isolates demonstrated substantial post-treatment growth recovery following transient heat and cold shock exposures, indicating resilience to rapid thermal fluctuations. Such tolerance is particularly relevant for microorganisms inhabiting steppe ecosystems, where abrupt day–night and seasonal temperature changes are common. The ability to withstand transient temperature extremes may therefore improve bacterial survival, persistence, and performance following field application where soil and climate conditions are often variable and unpredictable.

*Strains compatibility*. Often, bacterial consortiums consisting of several strains with different species and properties are more effective than individual strains. For example, a consortium of *Paenibacillus* sp. strain B2 and *Arthrobacter* sp. strain AA was more efficient to enhance wheat growth, resistance to *Septoria tritici* and tolerance to drought stress [62]. Furthermore, a consortium of *Paenibacillus* sp. Z15 and *Pseudomonas* sp. KR227 significantly enhanced root biomass, shoot length, and grain yield [57]. In our study, a consortium of *Pseudomonas* sp. ADL-13 and *Paenibacillus* sp. ZHS-9 would represent a complementary system, where both strains fulfill each other’s missing PGP traits (Table 3). For example, no IAA synthesis and N_2_-fixing ability of ADL-13 would be complemented with these characteristics of ZHS-9 while higher PSU and KSU values as well as siderophore production by ADL-13 would compensate these traits in ZHS-9. Nevertheless, the antagonistic activity of ADL-13 on ZHS-9 (Figure 7) prevented combination of these strains in one single consortium while both strains still can be applied as individual effective biofertilizers. These findings emphasize that complementary physiological traits alone are insufficient for successful consortium development. Mutual compatibility between candidate strains should also be verified, as antagonistic interactions may compromise consortium stability despite otherwise favorable plant growth-promoting characteristics.

*Morphological and structural characteristics*. SEM/TEM observations demonstrated cellular architecture of the isolates under baseline growth conditions. Similar SEM and TEM observations have been reported for both *Paenibacillus* and *Pseudomonas* species. For example, *Paenibacillus polymyxa* B7 and *Pseudomonas fluorescens* exhibited intact rod-shaped cells with well-preserved cellular morphology [93,94], which is consistent with the ultrastructural characteristics observed for ZHS-9 and ADL-13 in the present study.

## 5. Conclusions

Steppe-derived *Pseudomonas* sp. ADL-13 and *Paenibacillus* sp. ZHS-9 strains exhibited multiple plant growth-promoting traits and environmental stress resilience, including tolerance to glyphosate and various abiotic stresses. In wheat seedling bioassays, both strains significantly enhanced early plant development. Both strains showed phosphate and potassium solubilization and biofilm formation, while ADL-13 showed strong nutrient-solubilizing and siderophore-producing abilities, and ZHS-9 demonstrated putative nitrogen-fixing ability and IAA-like indolic compound production. Their ability to tolerate glyphosate and adverse environmental conditions, together with their plant growth-promoting activities, highlights their potential as candidate bioinoculants for sustainable agriculture and the development of environmentally friendly biofertilizers.

## Figures and Tables

**Table 1:** Isolation and identification of steppe-derived glyphosate-tolerant bacteria.

Strains	Sample and Isolation	Colony Diameter, mm			Genus Identification	
		0.1 g/L	0.5 g/L	1.0 g/L	16S rRNA	NCBI Accession Number
ZHS-4	SW-1-DP	4.07 ± 0.12	4.00 ± 0.00	3.40 ± 0.69	*Paenarthrobacter* sp.	PZ467928
ZHS-5	SW-1-DP	4.40 ± 0.53	4.00 ± 0.00	3.00 ± 0.00	*Paenarthrobacter* sp.	PZ467929
ZHS-6	SW-1-DP	4.57 ± 0.60	4.00 ± 0.00	3.33 ± 0.29	*Paenarthrobacter* sp.	PZ467930
ZHS-8	SW-1-DP	3.17 ± 0.29	3.73 ± 0.64	3.00 ± 0.00	*Paenibacillus* sp.	PZ467931
ZHS-9	SW-1-DP	2.17 ± 0.29	4.33 ± 1.53	3.17 ± 0.29	*Paenibacillus* sp.	PZ467932
AZ-1	SW-1-DP	3.33 ± 0.58	4.33 ± 1.53	4.00 ± 1.00	*Paenibacillus* sp.	PZ467923
AZ-2	SW-1-DP	3.00 ± 1.00	3.00 ± 0.00	3.33 ± 0.58	*Paenibacillus* sp.	PZ467924
AZ-3	SW-1-DP	3.50 ± 0.50	3.66 ± 2.08	3.66 ± 0.58	*Paenibacillus* sp.	PZ467925
ADL-13	SW-3-EC	3.17 ± 0.29	2.83 ± 0.29	2.83 ± 0.29	*Pseudomonas* sp.	PZ467926
ADL-20	SW-3-EC	2.67 ± 0.58	2.50 ± 0.50	2.00 ± 0.00	*Pseudomonas* sp.	PZ467927

Strain names (ADL, ZHS, and AZ) represent laboratory isolate codes assigned during strain isolation and selection. SW-1 and SW-3 are soil samples from two different cultivars of soft wheat. DP and EC are direct plating method and enrichment culture technique, respectively. Glyphosate concentration range included 0.1, 0.5, and 1.0 g/L. Glyphosate concentrations are expressed as g/L glyphosate acid equivalent (a.e.) delivered through the commercial formulation.

**Table 2:** Effect of cell-free culture filtrates (CCFs) of glyphosate-tolerant bacterial strains on wheat growth parameters.

Samples	Fresh-Mass, g	Number of Roots	Seedling Length, mm	Root Length, mm	Total Length, mm	Vigor Index
*Paenarthrobacter* and *Pseudomonas* strains						
WC	0.075 ± 0.00	4.30 ± 0.27	11.927 ± 0.09	9.039 ± 1.02	20.960 ± 0.94	196.1 ± 29.93
MC-I	0.062 ± 0.01	4.27 ± 0.15	11.186 ± 0.66	8.522 ± 0.31	19.700 ± 0.62	170.7 ± 30.55
ZHS-4	0.081 ± 0.01 ^m^	4.23 ± 0.53	12.896 ± 0.88	10.087 ± 0.84 ^m^	22.982 ± 1.71	190.0 ± 23.23
ZHS-5	0.078 ± 0.00	4.79 ± 0.18	13.249 ± 1.88	8.685 ± 0.27	21.934 ± 1.98	204.1 ± 10.86
ZHS-6	0.072 ± 0.01	4.55 ± 0.21	12.027 ± 3.60	8.213 ± 0.45	20.240 ± 4.04	175.9 ± 41.20
ADL-13	0.095± 0.01 ^wm^	4.74± 0.51	13.419 ± 1.55	9.880 ± 0.63	23.290 ± 0.94	224.9 ± 10.09
ADL-20	0.066 ± 0.01	4.27 ± 0.30	12.689 ± 2.78	8.875 ± 0.46	21.560 ± 2.89	202.3 ± 39.95
*Paenibacillus* strains						
WC	0.075 ± 0.00	4.30 ± 0.27	11.927 ± 0.09	9.039 ± 1.02	20.960 ± 0.94	196.1 ± 29.93
MC-II	0.085 ± 0.01	4.53 ± 0.35	12.071 ± 3.32	8.454 ± 1.24	20.525 ± 4.32	188.3 ± 14.53
AZ-1	0.081 ± 0.02	4.37 ± 0.12	14.223 ± 3.06	9.817 ± 1.62	24.040 ± 2.47	240.4 ± 24.74
AZ-2	0.071 ± 0.00	4.39 ± 0.25	13.409 ± 1.13	9.468 ± 1.37	22.877 ± 2.26	220.4 ± 12.57
AZ-3	0.097 ± 0.01 ^w^	4.59 ± 0.34	13.350 ± 0.85	9.556 ± 0.85	22.906 ± 0.88	221.8 ± 21.46
ZHS-8	0.091 ± 0.01	4.68 ± 0.33	14.972 ± 2.06	8.596 ± 1.40	23.569 ± 3.25	220.2 ± 34.63
ZHS-9	0.089 ± 0.00	4.57 ± 0.42	15.680 ± 0.96	7.893 ± 0.56	23.573 ± 0.45	235.7 ± 4.46

*Paenarthrobacter* (ZHS-4, ZHS-5, ZHS-6) and *Pseudomonas* (ADL-13, ADL-20) strains compared to the distilled WC and MC-I control (MM amended with L-alanine and 0.1 g/L tryptophan). *Paenibacillus* strains (AZ-1, AZ-2, AZ-3, ZHS-8, ZHS-9) compared to the same distilled water control as above (WC) and MC-II control (YEG amended with 0.1 g/L tryptophan). Superscript **^w^** indicates a significant difference from the water control (WC); **^m^** indicates a significant difference from the corresponding medium control (MC-I or MC-II); **^wm^** indicates a significant difference from both the WC and the corresponding medium control (MC-I or MC-II) (Dunnett’s test, *p* < 0.05). Significant differences (*p* < 0.05) are highlighted in bold. Values are mean ± SD.

**Table 3:** Plant growth-promoting profiles of steppe-derived ADL-13 and ZHS-9 strains.

PGPB Characteristics	*Pseudomonas* sp. ADL-13	*Paenibacillus* sp. ZHS-9
Glyphosate-tolerance, g/L	No inhibition (*p* > 0.05): 0.05–0.5 Promotion (*p* < 0.01): 1.0–2.0 Inhibition (*p* < 0.001): 5.0 g/L	No inhibition (*p* > 0.05): 0.5 Inhibition (*p* < 0.01): 1.0 Measurable growth (*p* < 0.01): 1.0–2.0
Wheat growth promotion, CCF, %	Fresh mass, (*p* < 0.05): 25.15–53.98 Root length, (*p* > 0.05): 9.30–15.93 Total length, (*p* > 0.05): 11.12–18.22 Vigor index, (*p* > 0.05): 14.72–31.76	Fresh mass, (*p* > 0.05): 17.10–4.24 Seedling length, (*p* > 0.05): 31.47–29.90 Total length, (*p* > 0.05): 12.44–14.85
Wheat growth promotion, LC, %	Fresh mass, (*p* < 0.05): 37.33–43.14 Root length, (*p* < 0.05): 36.67–32.13 Total length, (*p* < 0.05): 22.31–28.42 Vigor index, (*p* < 0.05): 22.31–43.13	Fresh mass, (*p* ≤ 0.05): 28.25–17.33 Seedling length, (*p* < 0.05): 17.64–20.49 Total length, (*p* < 0.05): 16.45–14.35
P, CaHPO_4_, PSI	1.99 ± 0.34	1.28 ± 0.07
P, Ca_3_(PO_4_)_2_, PSI	1.13 ± 0.07	−
K, KSI	1.11 ± 0.05	1.20 ± 0.05
IAA-like indolic compounds, µg/mL	−	20.97 ± 12.90
Siderophores, PSU	SMM + Succinic acid: 33.93 ± 1.93	YEG: 3.40 ± 0.89
	SMM + Glucose: 63.05 ± 2.00	
	YEG: 13.35 ± 2.68	
NH_3_, µM	PW: 26.43 ± 4.37	PW: −
	YEG: 11.53 ± 1.01	YEG: 8.31 ± 0.76
Putative N_2_-fixing ability	−	+
OA secretion	+	±
Biofilm formation	+	+

Wheat promotion: values represent percentage differences in the indicated growth parameters relative to the two controls, with WC as the first value and MC-I (for ADL-13) or MC-II (for ZHS-9) as the second value. Growth parameters marked as *p* < 0.05 indicate statistically significant promotion relative to both controls (including the borderline value of *p* = 0.0579 for ZHS-9 fresh mass compared with MC-II). Parameters marked as *p* > 0.05 indicate that statistical significance was not reached against one or both controls. Siderophore production is expressed as percentage siderophore units (PSU, %), whereas ammonia production is expressed as µM NH_3_. Putative N_2_-fixing ability indicates the potential for N_2_ fixation inferred from the applied assay and does not confirm direct nitrogen fixation. The signs “+” and “−” represent positive and negative indicators, respectively; the sign “±” indicates presumptive OA secretion.

**Table 4:** Effects of *Paenibacillus* and *Pseudomonas* strains on growth parameters of wheat and barley seedlings.

Strain	Crop	Parameter Measured	Effect, %	References
**Biomass-Related Traits (FW—Fresh Weight; DW—Dry Weight, as Reported in the References)**				
*Pseudomonas* sp. ADL-13	Wheat	Fresh mass	25.15–43.14%	Present study
*Paenibacillus* sp. ZHS-9	Wheat	Fresh mass	17.10–28.25%	
*Paenibacillus* strain 130	Wheat	FW; DW	16.7% FW; 20.4% DW	[60]
*Paenibacillus* strain 122	Wheat	FW; DW	4.2% FW; 3.7% DW	
*Pseudomonas* sp. PNS-4	Wheat	FW; DW	45.9% FW; 51.6% DW	[61]
*Pseudomonas* sp. PNS-6	Wheat	FW; DW	49.6% FW; 61.3% DW	
**Root** **Traits**				
*Pseudomonas* sp. ADL-13	Wheat	Root length	15.93–36.67%	Present study
*Paenibacillus* sp. B2	Wheat	Root biomass	67%	[62]
*Paenibacillus alvei* NAS6G-6	Wheat	Root FW; Root DW; Total root length	125.0% FW; 33.3% DW; 16.5% length	[63]
*Paenibacillus nicotianae* AFI2	Wheat	Root length	2.8%	[64]
*Paenibacillus nicotianae* AFI2	Wheat	Root length	33.9%	
*Paenibacillus polymyxa* SGK2	Durum wheat	Root DW (restoration)	90%	[65]
*Paenibacillus* strain 130	Wheat	Root length	11.1%	[60]
*Paenibacillus* strain 122	Wheat	Root length	1.6%	
*Pseudomonas* sp. PNS-4	Wheat	Root length	46.2%	[61]
*Pseudomonas* sp. PNS-6	Wheat	Root length	53.7%	
*Paenibacillus polymyxa* RC05	Barley	Root length	6.0%	[66]
*Paenibacillus polymyxa* RC05	Barley	Root FW	12.0%	
*Pseudomonas putida* RC06	Barley	Root length	5.4%	[66]
*Pseudomonas putida* RC06	Barley	Root FW	8.0%	
*Pseudomonas fluorescens* CHA0	Barley	Root-associated FW	51–53% root FW	[67]
*Paenibacillus sp.* Z15 + *Pseudomonas sp.* KR227	Barley	Root FW	120.1%	[57]
**Shoot and Seedling Growth**				
*Paenibacillus* sp. ZHS-9	Wheat	Seedling length	17.64–31.47%	Present study
*Pseudomonas* sp. ADL-13	Wheat	Total length	11.12–28.42%	
*Paenibacillus* sp. ZHS-9	Wheat	Total length	12.44–16.45%	
*Paenibacillus alvei* NAS6G-6	Wheat	Shoot DW; Shoot FW	68.0%; 85.0%	[63]
*Paenibacillus nicotianae* AFI2	Wheat	Shoot length; Shoot FW	21.1%; 30.1%	[64]
*Paenibacillus nicotianae* AFI2	Wheat	Shoot length; shoot FW	6.1% length; 34.7% FW;	
*Paenibacillus* strain 130	Wheat	Shoot length	16.0%	[60]
*Paenibacillus* strain 122	Wheat	Shoot length	4.0%	
*Pseudomonas* sp. PNS-4	Wheat	Shoot length	26.5%	[61]
*Pseudomonas sp.* PNS-6	Wheat	Shoot length	20.4%	
*Pseudomonas fluorescens* WB1–7	Wheat	Seedling length; seedling DW	19.0%; 16.8% DW	[68]
*Pseudomonas fluorescens* PGU2–79	Wheat	Seedling length; seedling DW	23.9%; 47.8% DW	
*Paenibacillus polymyxa* RC05	Barley	Shoot FW	36.1%	[66]
*Pseudomonas putida* RC06	Barley	Shoot FW	30.1%	
*Pseudomonas fluorescens* CHA0	Barley	Shoot FW	2.9%	[67]
*Paenibacillus sp.* Z15 + *Pseudomonas sp.* KR227	Barley	Shoot length	7.8%	[57]
**Vigor-Related Traits**				
*Pseudomonas* sp. ADL-13	Wheat	Vigor index	14.72–43.13%	Present study
*Pseudomonas fluorescens* WB1–7	Wheat	Seed vigor	81.1%	[68]
*Pseudomonas fluorescens* PGU2–79	Wheat	Seed vigor	63.7%	

Literature values serve as a general contextual benchmark. Direct quantitative comparisons across studies should be interpreted with caution due to differences in cultivars, assay conditions, and methodology.

## Data Availability

The 16S rRNA gene sequences generated in this study are available in the NCBI database under accession numbers PZ467923-PZ467932. The *gyrA1* and *gyrA2* sequences of *Paenibacillus* sp. ZHS-9 and the *rpoB* and *rpoD* sequences of *Pseudomonas* sp. ADL-13 have been deposited in the NCBI GenBank database under accession numbers PZ771143, PZ771144, PZ771145, and PZ771146, respectively. Further generated data of this study are available from the corresponding author under reasonable request.

## Supplementary Materials

The following supporting information can be downloaded at: https://www.mdpi.com/article/10.3390/microorganisms14102371/s1, Supplementary Table S1. Characteristics and glyphosate treatment history of agricultural steppe soils used for the isolation of glyphosate-tolerant bacteria; Supplementary Table S2. List of gene and genome accessions used in this study; Supplementary Figure S1. Organic acid (OA) secretion of glyphosate-tolerant *Pseudomonas* sp. ADL-13 (A) and *Paenibacillus* sp. ZHS-9 (B). Red arrowheads indicate the outer boundary of the prominent halo for ADL-13 and the faint, localized clearing of putative OA secretion for ZHS-9; Supplementary Figure S2. Biofilm formation of glyphosate-tolerant *Pseudomonas* sp. ADL-13 and *Paenibacillus* sp. ZHS-9. Higher OD590 values relative to the non-inoculated control indicated greater biofilm-forming capacity under the assay conditions. Different letters indicate significant differences according to one-way ANOVA followed by Tukey’s HSD test (*p* < 0.05). Bars represent the mean of six replicates (*n* = 6), and error bars represent standard deviations (SD); Supplementary Figure S3. The growth of glyphosate-tolerant *Pseudomonas* sp. ADL-13 (A) and *Paenibacillus* sp. ZHS-9 (B) on nitrogen-free Ashby’s medium (NH4Cl-) and Ashby’s medium supplied with 0.27 g/L NH4Cl (NH4Cl+) as the control. Growth on nitrogen-free medium as an indicator of putative N-fixing potential; Supplementary Figure S4. SEM image of *Pseudomonas* sp. ADL-13 strain as shown in Figure 8A (uncropped original image); Supplementary Figure S5. TEM image of *Pseudomonas* sp. ADL-13 strain as shown in Figure 8B (uncropped original image); Supplementary Figure S6. SEM image of *Paenibacillus* sp. ZHS-9 strain as shown in Figure 8C (uncropped original image); Supplementary Figure S7. TEM image of *Paenibacillus* sp. ZHS-9 strain as shown in Figure 8D (uncropped original image).

## References

[1] von Mérey G., Manson P.S., Mehrsheikh A., Sutton P., Levine S.L. (2016). Glyphosate and Aminomethylphosphonic Acid Chronic Risk Assessment for Soil Biota. Environ. Toxicol. Chem..

[2] Duke S.O., Powles S.B. (2008). Glyphosate: A Once-in-a-Century Herbicide. Pest Manag. Sci..

[3] Kanissery R., Gairhe B., Kadyampakeni D., Batuman O., Alferez F. (2019). Glyphosate: Its Environmental Persistence and Impact on Crop Health and Nutrition. Plants.

[4] De Roos A.J., Blair A., Rusiecki J.A., Hoppin J.A., Svec M., Dosemeci M., Sandler D.P., Alavanja M.C. (2005). Cancer Incidence among Glyphosate-Exposed Pesticide Applicators in the Agricultural Health Study. Environ. Health Perspect..

[5] Gillezeau C., van Gerwen M., Shaffer R.M., Rana I., Zhang L., Sheppard L., Taioli E. (2019). The Evidence of Human Exposure to Glyphosate: A Review. Environ. Health.

[6] Leon M.E., Schinasi L.H., Lebailly P., Beane Freeman L.E., Nordby K.-C., Ferro G., Monnereau A., Brouwer M., Tual S., Baldi I. (2019). Pesticide Use and Risk of Non-Hodgkin Lymphoid Malignancies in Agricultural Cohorts from France, Norway and the USA: A Pooled Analysis from the AGRICOH Consortium. Int. J. Epidemiol..

[7] Bartholomew S.K., Winslow W., Sharma R., Pathak K.V., Tallino S., Judd J.M., Leon H., Turk J., Pirrotte P., Velazquez R. (2024). Glyphosate Exposure Exacerbates Neuroinflammation and Alzheimer’s Disease-like Pathology despite a 6-Month Recovery Period in Mice. J. Neuroinflamm..

[8] Etesami H., Maheshwari D.K. (2018). Use of Plant Growth Promoting Rhizobacteria (PGPRs) with Multiple Plant Growth Promoting Traits in Stress Agriculture: Action Mechanisms and Future Prospects. Ecotoxicol. Environ. Saf..

[9] Aislabie J., Deslippe J.R., Dymond J.R. (2013). Soil Microbes and Their Contribution to Soil Services. Ecosystem Services in New Zealand: Conditions and Trends.

[10] Fiodor A., Singh S., Pranaw K. (2021). The Contrivance of Plant Growth Promoting Microbes to Mitigate Climate Change Impact in Agriculture. Microorganisms.

[11] Glick B.R., Cheng Z., Czarny J., Duan J. (2007). Promotion of Plant Growth by ACC Deaminase-Producing Soil Bacteria. Eur. J. Plant Pathol..

[12] Glick B.R. (2012). Plant Growth-Promoting Bacteria: Mechanisms and Applications. Scientifica.

[13] Basu A., Prasad P., Das S.N., Kalam S., Sayyed R.Z., Reddy M.S., Enshasy H. (2021). El Plant Growth Promoting Rhizobacteria (Pgpr) as Green Bioinoculants: Recent Developments, Constraints, and Prospects. Sustainability.

[14] Fanai A., Bohia B., Lalremruati F., Lalhriatpuii N., Lalrokimi, Lalmuanpuii R., Singh P.K., Zothanpuia (2024). Plant Growth Promoting Bacteria (PGPB)-Induced Plant Adaptations to Stresses: An Updated Review. PeerJ.

[15] Mohy-Ud-Din W., Akhtar M.J., Bashir S., Asghar H.N., Nawaz M.F., Chen F. (2023). Isolation of Glyphosate-Resistant Bacterial Strains to Improve the Growth of Maize and Degrade Glyphosate under Axenic Condition. Agriculture.

[16] Mohy-Ud-Din W., Chen F., Bashir S., Akhtar M.J., Asghar H.N., Ali Q., Hassan W., Yasin S., Raza M.H., Choudary Z.M. (2025). Enhancing Maize Yield and Antioxidant Capacity with Glyphosate-Resilient Rhizobacteria in Glyphosate Contaminated Soil. BMC Plant Biol..

[17] Bhattacharyya P.N., Jha D.K. (2012). Plant Growth-Promoting Rhizobacteria (PGPR): Emergence in Agriculture. World J. Microbiol. Biotechnol..

[18] Ghadamgahi F., Tarighi S., Taheri P., Saripella G.V., Anzalone A., Kalyandurg P.B., Catara V., Ortiz R., Vetukuri R.R. (2022). Plant Growth-Promoting Activity of *Pseudomonas aeruginosa* FG106 and Its Ability to Act as a Biocontrol Agent against Potato, Tomato and Taro Pathogens. Biology.

[19] Singh P., Singh R.K., Zhou Y., Wang J., Jiang Y., Shen N., Wang Y., Yang L., Jiang M. (2022). Unlocking the Strength of Plant Growth Promoting *Pseudomonas* in Improving Crop Productivity in Normal and Challenging Environments: A Review. J. Plant Interact..

[20] Dobrzyński J., Naziębło A. (2024). *Paenibacillus* as a Biocontrol Agent for Fungal Phytopathogens: Is *P. polymyxa* the Only One Worth Attention?. Microb. Ecol..

[21] Dobrzyński J., Kulkova I. (2025). *Paenibacillus peoriae*: Current Knowledge and Agricultural Biotechnology Potential of a Close Relative of *P. polymyxa*. Antonie Leeuwenhoek.

[22] Zhaksybek Z., Sattarova A., Akimbekova A., Shamukhan A., Rukavitsina I., Abeldenov S., Zhumakayev A.R. (2026). Integrative Exploration of *Paenibacillus* sp. JSM-10 as a Potential Multi-Stress-Tolerant Microbial Inoculant for Sustainable Agriculture. Int. J. Mol. Sci..

[23] Zhumakayev A.R., Vörös M., Szekeres A., Rakk D., Vágvölgyi C., Szűcs A., Kredics L., Škrbić B.D., Hatvani L. (2021). Comprehensive Characterization of Stress Tolerant Bacteria with Plant Growth-Promoting Potential Isolated from Glyphosate-Treated Environment. World J. Microbiol. Biotechnol..

[24] Zamule S.M., Dupre C.E., Mendola M.L., Widmer J., Shebert J.A., Roote C.E., Das P. (2021). Bioremediation Potential of Select Bacterial Species for the Neonicotinoid Insecticides, Thiamethoxam and Imidacloprid. Ecotoxicol. Environ. Saf..

[25] Muyzer G., De Waal E.C., Uitterlinden A.G. (1993). Profiling of Complex Microbial Populations by Denaturing Gradient Gel Electrophoresis Analysis of Polymerase Chain Reaction-Amplified Genes Coding for 16S RRNA. Appl. Environ. Microbiol..

[26] Kocsubé S., Papp D.A., Farkas A., Huynh T., Allaga H., Szekeres A., Vágvölgyi C., Ámon J., Hamari Z., Varga M. (2026). *Pseudomonas rhizozeae* sp. Nov., Originated from Rhizosphere Soil of Corn Field. Sci. Rep..

[27] Katoh K., Standley D.M. (2013). MAFFT Multiple Sequence Alignment Software Version 7: Improvements in Performance and Usability. Mol. Biol. Evol..

[28] Vaidya G., Lohman D.J., Meier R. (2011). SequenceMatrix: Concatenation Software for the Fast Assembly of Multi-Gene Datasets with Character Set and Codon Information. Cladistics.

[29] Kalyaanamoorthy S., Minh B.Q., Wong T.K.F., von Haeseler A., Jermiin L.S. (2017). ModelFinder: Fast Model Selection for Accurate Phylogenetic Estimates. Nat. Methods.

[30] Minh B.Q., Schmidt H.A., Chernomor O., Schrempf D., Woodhams M.D., von Haeseler A., Lanfear R. (2020). IQ-TREE 2: New Models and Efficient Methods for Phylogenetic Inference in the Genomic Era. Mol. Biol. Evol..

[31] Hoang D.T., Chernomor O., von Haeseler A., Minh B.Q., Vinh L.S. (2018). UFBoot2: Improving the Ultrafast Bootstrap Approximation. Mol. Biol. Evol..

[32] Vágvölgyi C., Magyar K., Papp T., Palágyi Z., Ferenczy L., Nagy Á. (1996). Value of Substrate Utilization Data for Characterization of Mucor Isolates. Can. J. Microbiol..

[33] Zhumakayev A.R., Varga M., Vörös M., Kocsubé S., Ramteke P.W., Szekeres A., Vágvölgyi C., Hatvani L., Marik T. (2022). Characterization of the Antagonistic Potential of the Glyphosate-Tolerant *Pseudomonas resinovorans* SZMC 25872 Strain against the Plant Pathogenic Bacterium *Agrobacterium tumefaciens*. Front. Plant Sci..

[34] Sasani M., Piri R., Zamani F., Fazeli-Nasab B. (2026). Seed Biopriming with Plant Growth Promoting Bacteria Improves Germination and Seedling Performance in Wheat (*Triticum aestivum* L.). BMC Plant Biol..

[35] Pikovskaya R.I. (1948). Mobilization of Phosphorus in Soil in Connection with the Vital Activity of Some Microbial Species. Microbiology.

[36] Teles E.A.P., Xavier J.F., Arcênio F.S., Amaya R.L., Gonçalves J.V.S., Rouws L.F.M., Zonta E., Coelho I.S. (2024). Characterization and Evaluation of Potential Halotolerant Phosphate Solubilizing Bacteria from *Salicornia fruticosa* Rhizosphere. Front. Plant Sci..

[37] Bric J.M., Bostock R.M., Silverstone S.E. (1991). Rapid in Situ Assay for Indoleacetic Acid Production by Bacteria Immobilized on a Nitrocellulose Membrane. Appl. Environ. Microbiol..

[38] Schwyn B., Neilands J.B. (1987). Universal Chemical Assay for the Detection and Determination of Siderophores. Anal. Biochem..

[39] Meyer J.M., Abdallah M.A. (1978). The Fluorescent Pigment of *Pseudomonas fluorescens*: Biosynthesis, Purification and Physicochemical Properties. Microbiology.

[40] Payne S.M. (1994). Detection, isolation, and characterization of siderophores. Methods Enzymol..

[41] Rashad Y.M., Hafez M., Rashad M. (2023). Diazotrophic Azotobacter Salinestris YRNF3: A Probable Calcite-Solubilizing Bio-Agent for Improving the Calcareous Soil Properties. Sci. Rep..

[42] Kerekes E.B., Deák É., Takó M., Tserennadmid R., Petkovits T., Vágvölgyi C., Krisch J. (2013). Anti-Biofilm Forming and Anti-Quorum Sensing Activity of Selected Essential Oils and Their Main Components on Food-Related Micro-Organisms. J. Appl. Microbiol..

[43] Aslam A., Ahmad Zahir Z., Asghar H.N., Shahid M. (2018). Effect of Carbonic Anhydrase-Containing Endophytic Bacteria on Growth and Physiological Attributes of Wheat under Water-Deficit Conditions. Plant Prod. Sci..

[44] Taffner J., Laggner O., Wolfgang A., Coyne D., Berg G. (2020). Exploring the Microbiota of East African Indigenous Leafy Greens for Plant Growth, Health, and Resilience. Front. Microbiol..

[45] Nielsen C.K., Kjems J., Mygind T., Snabe T., Meyer R.L. (2016). Effects of Tween 80 on Growth and Biofilm Formation in Laboratory Media. Front. Microbiol..

[46] Britton H.T.S., Robinson R.A. (1931). CXCVIII.—Universal buffer solutions and the dissociation constant of veronal. J. Chem. Soc..

[47] Fox J., Weisberg S. (2018). An R Companion to Applied Regression.

[48] Signorell A. (2025). DescTools: Tools for Descriptive Statistics, version 0.99.60.

[49] de Mendiburu F. (2023). agricolae: Statistical Procedures for Agricultural Research.

[50] Wickham H. (2016). Ggplot2: Elegant Graphics for Data Analysis.

[51] Pedersen T. (2025). patchwork: The Composer of Plots.

[52] Doolotkeldieva T., Bobusheva S., Konurbaeva M. (2025). The Bacterial Species’ Degradation Activities at Maximum Threshold Doses of Glyphosate across Different pH Levels and Temperature Glyphosate Biodegradation by Soil Bacteria at High Doses under Variable pH and Temperature. Front. Microbiol..

[53] Ahuja M., Kumar L., Kumar K., Shingatgeri V.M., Kumar S. (2024). Glyphosate: A Review on Its Widespread Prevalence and Occurrence across Various Systems. Environ. Sci. Adv..

[54] Silva B.C., de Souza B.G., Bertolazi A.A., Pêgos A.R., Cruz C., Melo J., Babalola O.O., Krohling C.A., Ramos A.C. (2025). Isolation and Characterization of Glyphosate-Tolerant Bacteria from a Local Coffee Plantation in Espírito Santo, Brazil. ACS Agric. Sci. Technol..

[55] Batykova Z., Kistaubayeva A., Abdulzhanova M., Doktyrbay G., Saidullayeva L., Baimirzayeva Z., Turaliyeva M., Ibraimova Z. (2026). Multifunctional Maize Rhizobacteria: Isolation, Characterization and Prospects for Sustainable Agriculture. Int. J. Plant Biol..

[56] Ajijah N., Fiodor A., Pandey A.K., Rana A., Pranaw K. (2023). Plant Growth-Promoting Bacteria (PGPB) with Biofilm-Forming Ability: A Multifaceted Agent for Sustainable Agriculture. Diversity.

[57] Dobrzyński J., Naziębło A., Kulkova I., Szpytma M., Antosik A., Sitarek-Andrzejczyk M., Wróbel B. (2026). *Paenibacillus–Pseudomonas* Consortium Improves Barley Performance with Minimal Impact on Native Rhizobacterial Community. Microorganisms.

[58] Backer R., Rokem J.S., Ilangumaran G., Lamont J., Praslickova D., Ricci E., Subramanian S., Smith D.L. (2018). Plant Growth-Promoting Rhizobacteria: Context, Mechanisms of Action, and Roadmap to Commercialization of Biostimulants for Sustainable Agriculture. Front. Plant Sci..

[59] Trivedi P., Leach J.E., Tringe S.G., Sa T., Singh B.K. (2020). Plant–Microbiome Interactions: From Community Assembly to Plant Health. Nat. Rev. Microbiol..

[60] Zhao L., Xu Y., Lai X.-H., Shan C., Deng Z., Ji Y. (2015). Screening and Characterization of Endophytic *Bacillus* and *Paenibacillus* Strains from Medicinal Plant *Lonicera Japonica* for Use as Potential Plant Growth Promoters. Braz. J. Microbiol..

[61] Iqbal A., Hasnain S. (2013). Auxin Producing *Pseudomonas* Strains: Biological Candidates to Modulate the Growth of *Triticum aestivum* Beneficially. Am. J. Plant Sci..

[62] Samain E., Ernenwein C., Aussenac T., Selim S. (2022). Effective and durable systemic wheat-induced resistance by a plant-growth-promoting rhizobacteria consortium of Paenibacillus sp. strain B2 and Arthrobacter spp. strain AA against Zymoseptoria tritici and drought stres. Physiol. Mol. Plant Pathol.

[63] Hassen A.I., Labuschagne N. (2010). Root Colonization and Growth Enhancement in Wheat and Tomato by Rhizobacteria Isolated from the Rhizoplane of Grasses. World J. Microbiol. Biotechnol..

[64] Pishchik V.N., Filippova P.S., Mirskaya G.V., Khomyakov Y.V., Vertebny V.E., Dubovitskaya V.I., Ostankova Y.V., Semenov A.V., Chakrabarty D., Zuev E.V. (2021). Epiphytic PGPB *Bacillus megaterium* AFI1 and *Paenibacillus nicotianae* AFI2 Improve Wheat Growth and Antioxidant Status under Ni Stress. Plants.

[65] Lounaci L., Guemouri-Athmani S., Boureghda H., Achouak W., Heulin T. (2017). Suppression of Crown and Root Rot of Wheat by the Rhizobacterium *Paenibacillus polymyxa*. Phytopathol. Mediterr..

[66] Canbolat M.Y., Barik K., Çakmakçi R., Şahin F. (2006). Effects of Mineral and Biofertilizers on Barley Growth on Compacted Soil. Acta Agric. Scand. Sect. B-Soil Plant Sci..

[67] Henkes G.J., Jousset A., Bonkowski M., Thorpe M.R., Scheu S., Lanoue A., Schurr U., Röse U.S.R. (2011). *Pseudomonas fluorescens* CHA0 Maintains Carbon Delivery to *Fusarium graminearum*-Infected Roots and Prevents Reduction in Biomass of Barley Shoots through Systemic Interactions. J. Exp. Bot..

[68] Safari D., Jamali F., Nooryazdan H., Bayat F. (2018). Evaluation of ACC Deaminase Producing *Pseudomonas fluorescens* Strains for Their Effects on Seed Germination and Early Growth of Wheat Under Salt Stress. Aust. J. Crop Sci..

[69] Khalid A., Arshad M., Zahir Z.A. (2004). Screening Plant Growth-promoting Rhizobacteria for Improving Growth and Yield of Wheat. J. Appl. Microbiol..

[70] Olanrewaju O.S., Glick B.R., Babalola O.O. (2017). Mechanisms of Action of Plant Growth Promoting Bacteria. World J. Microbiol. Biotechnol..

[71] Chattaraj S., Samantaray A., Ganguly A., Thatoi H. (2025). Employing Plant Growth-Promoting Rhizobacteria for Abiotic Stress Mitigation in Plants: With a Focus on Drought Stress. Discov. Appl. Sci..

[72] Fernández-Pastrana V.M., González-Reguero D., Robas-Mora M., Penalba-Iglesias D., Alonso-Torreiro P., Probanza A., Jiménez-Gómez P.A. (2025). Biotechnological Test of Plant Growth-Promoting Bacteria Strains for Synthesis of Valorized Wastewater as Biofertilizer for Silvicultural Production of Holm Oak (*Quercus ilex* L.). Plants.

[73] Fouzia A., Allaoua S., Hafsa C.-S., Mostefa G. (2015). Plant Growth Promoting and Antagonistic Traits of Indigenous Fluorescent *Pseudomonas* spp. Isolated from Wheat Rhizosphere and *A. halimus* Endosphere. Eur. Sci. J. ESJ.

[74] Behera B.C., Singdevsachan S.K., Mishra R.R., Dutta S.K., Thatoi H.N. (2014). Diversity, Mechanism and Biotechnology of Phosphate Solubilising Microorganism in Mangrove—A Review. Biocatal. Agric. Biotechnol..

[75] Sarker A., Talukder N.M., Islam T. (2014). Phosphate Solubilizing Bacteria Promote Growth and Enhance Nutrient Uptake by Wheat. Plant Sci..

[76] Chow L.C. (2001). Solubility of Calcium Phosphates. Octacalcium Phosphate.

[77] Ou K., He X., Cai K., Zhao W., Jiang X., Ai W., Ding Y., Cao Y. (2022). Phosphate-Solubilizing *Pseudomonas* sp. Strain WS32 Rhizosphere Colonization-Induced Expression Changes in Wheat Roots. Front. Microbiol..

[78] Ruan J., Mao B., Zhuo Y., Jiang Q. (2026). Advances in Mechanisms and Applications of Potassium-Solubilizing Bacteria. Int. J. Biol. Life Sci..

[79] Ahmed E., Holmström S.J.M. (2014). Siderophores in Environmental Research: Roles and Applications. Microb. Biotechnol..

[80] Zuluaga M.Y.A., Cardarelli M., Rouphael Y., Cesco S., Pii Y., Colla G. (2023). Iron Nutrition in Agriculture: From Synthetic Chelates to Biochelates. Sci. Hortic..

[81] Zhang Y., Ren J., Wang W., Chen B., Li E., Chen S. (2020). Siderophore and Indolic Acid Production by *Paenibacillus Triticisoli* BJ-18 and Their Plant Growth-Promoting and Antimicrobe Abilities. PeerJ.

[82] de Oliveira D.A., Ferreira S.D.C., Carrera D.L.R., Serrão C.P., Callegari D.M., Barros N.L.F., Coelho F.M., de Souza C.R.B. (2021). Characterization of *Pseudomonas* Bacteria of *Piper tuberculatum* Regarding the Production of Potentially Bio-Stimulating Compounds for Plant Growth. Acta Amaz..

[83] Ali A., Jabeen N., Farruhbek R., Chachar Z., Laghari A.A., Chachar S., Ahmed N., Ahmed S., Yang Z. (2025). Enhancing Nitrogen Use Efficiency in Agriculture by Integrating Agronomic Practices and Genetic Advances. Front. Plant Sci..

[84] Liu X., Li Q., Li Y., Guan G., Chen S. (2019). *Paenibacillus* Strains with Nitrogen Fixation and Multiple Beneficial Properties for Promoting Plant Growth. PeerJ.

[85] Weselowski B., Nathoo N., Eastman A.W., MacDonald J., Yuan Z.-C. (2016). Isolation, Identification and Characterization of *Paenibacillus polymyxa* CR1 with Potentials for Biopesticide, Biofertilization, Biomass Degradation and Biofuel Production. BMC Microbiol..

[86] Tamilselvi S.M., Thiyagarajan C., Uthandi S. (2016). Calcite Dissolution by *Brevibacterium* sp. SOTI06: A Futuristic Approach for the Reclamation of Calcareous Sodic Soils. Front. Plant Sci..

[87] Rudrappa T., Biedrzycki M.L., Bais H.P. (2008). Causes and Consequences of Plant-Associated Biofilms. FEMS Microbiol. Ecol..

[88] Radhakrishnan N., Krishnasamy C. (2024). Isolation and Characterization of Salt-Stress-Tolerant Rhizosphere Soil Bacteria and Their Effects on Plant Growth-Promoting Properties. Sci. Rep..

[89] Nakbanpote W., Panitlurtumpai N., Sangdee A., Sakulpone N., Sirisom P., Pimthong A. (2014). Salt-Tolerant and Plant Growth-Promoting Bacteria Isolated from Zn/Cd Contaminated Soil: Identification and Effect on Rice under Saline Conditions. J. Plant Interact..

[90] Wang C., Pei J., Li H., Zhu X., Zhang Y., Wang Y., Li W., Wang Z., Liu K., Du B. (2024). Mechanisms on Salt Tolerant of *Paenibacillus polymyxa* SC2 and Its Growth-Promoting Effects on Maize Seedlings under Saline Conditions. Microbiol. Res..

[91] Bouremani N., Cherif-Silini H., Silini A., Rabhi N.E.H., Bouket A.C., Belbahri L. (2024). Osmotolerant Plant Growth Promoting Bacteria Mitigate Adverse Effects of Drought Stress on Wheat Growth. AIMS Microbiol..

[92] Reitermayer D., Kafka T.A., Lenz C.A., Vogel R.F. (2018). Interrelation between Tween and the Membrane Properties and High Pressure Tolerance of *Lactobacillus plantarum*. BMC Microbiol..

[93] Zhao T., Ma J., Lin M., Gao C., Zhao Y., Li X., Sun W. (2024). Isolation and Characterization of *Paenibacillus polymyxa* B7 and Inhibition of *Aspergillus tubingensis* A1 by Its Antifungal Substances. Int. J. Mol. Sci..

[94] Wang D., Liu Y., Han T., Zhang C., Liu D., Zhang J. (2025). Anti-*Pseudomonas fluorescens* Mechanism of Plantaricin Q7 from *Lactiplantibacillus plantarum* Q7 Based on Proteomics. Food Sci. Anim. Prod..

