# Supplementary material for: Steppe-Derived Glyphosate-Tolerant Pseudomonas sp. ADL-13 and Paenibacillus sp. ZHS-9 Strains with Plant Growth-Promoting Traits and Broad Abiotic Stress Tolerance
Source: Microorganisms. 2026 Oct 16;14(10):2371. doi: 10.3390/microorganisms14102371 (PMC13707483; doi:10.3390/microorganisms14102371)
Supplement: Supplementary file 1 [file microorganisms-14-02371-s001.zip › microorganisms-4573836-supplementary.pdf]

1.1 Supplementary Tables

Supplementary Table S1. Characteristics and glyphosate treatment history of agricultural steppe soils used for the isolation of glyphosate-tolerant bacteria.

**Soil sample code, soil type, and collection date**	**Previous crop**	**Crop and cultivar**	**Glyphosate treatment**	
			**Treatment type**	**Date**
SW-1,<br>Calcaric Haplic Chernozem, 22.08.2024	Fallow<br>(No-Till)	*Triticum aestivum* L.<br>“Astana”	Pre-sowing chemical treatment with “Uragan Forte” at 2 L/ha	20.05.2023
			Chemical weeding of no-till fallow with “Uragan Forte” at 2 L/ha	04.08.2023
			Pre-sowing chemical treatment with “Pharaon Gold” at 2 L/ha	29.05.2024
SW-3,<br>Haplic Chernozem, 21.08.2024	*Triticum aestivum* L.	*Triticum aestivum* L.<br>*“Element 22”*	Pre-sowing chemical treatment with “Smerch” at 2 L/ha	early June, 2024

SW-1 and SW-3 are soil samples from two different cultivars of soft wheat.

Supplementary Table S2. List of gene and genome accessions used in this study.

**Species**	**Isolate**	**Accession**
Paenibacillus brasilensis	KACC 13842	GCF_009363115.1
Paenibacillus farraposensis	CCM 9147	GCA_042665895.1
Paenibacillus jamilae	NS115	GCA_001477135.1
Paenibacillus kribbensis	AM49	GCF_002240415.1
Paenibacillus maysiensis	1-49	GCF_000520815.1
Paenibacillus ottowii	MS2379	GCA_006874425.1
Paenibacillus peoriae	KCTC 3763	GCA_000236805.2
Paenibacillus polymyxa	DSM 36	GCA_015710975.1
Paenibacillus terrae	HPL-003	GCA_007680025.1
Pseudomonas brenneri	DSM 15294	GCA_007858285.1
Pseudomonas endophytica	BSTT44	GCA_001411475.1
Pseudomonas gessardii	LMG 21604	GCA_900101185.1
Pseudomonas helleri	DSM 29165	GCA_001043025.1
Pseudomonas hygromyciniae	SDM007	GCA_016925675.1
Pseudomonas karstica	CCM 7891	GCA_009707515.1
Pseudomonas mucidolens	LMG 2223	GCA_900106045.1
Pseudomonas proteolytica	LMG 22710	GCA_900105955.1
Pseudomonas shahriarae	SWRI52	GCF_014268455.2
Pseudomonas spelaei	CCM 7893	GCA_042659925.1
Pseudomonas yamanorum	LMG 27247	GCA_900105735.1

The 16S rRNA gene sequences generated in this study are available in the NCBI database under accession numbers PZ467923-PZ467932. The gyrA1 and gyrA2 sequences of *Paenibacillus* sp. ZHS-9 and the rpoB and rpoD sequences of *Pseudomonas* sp. ADL-13 have been deposited in the NCBI GenBank database under accession numbers PZ771143, PZ771144, PZ771145, and PZ771146, respectively.

1.2 Supplementary Figures

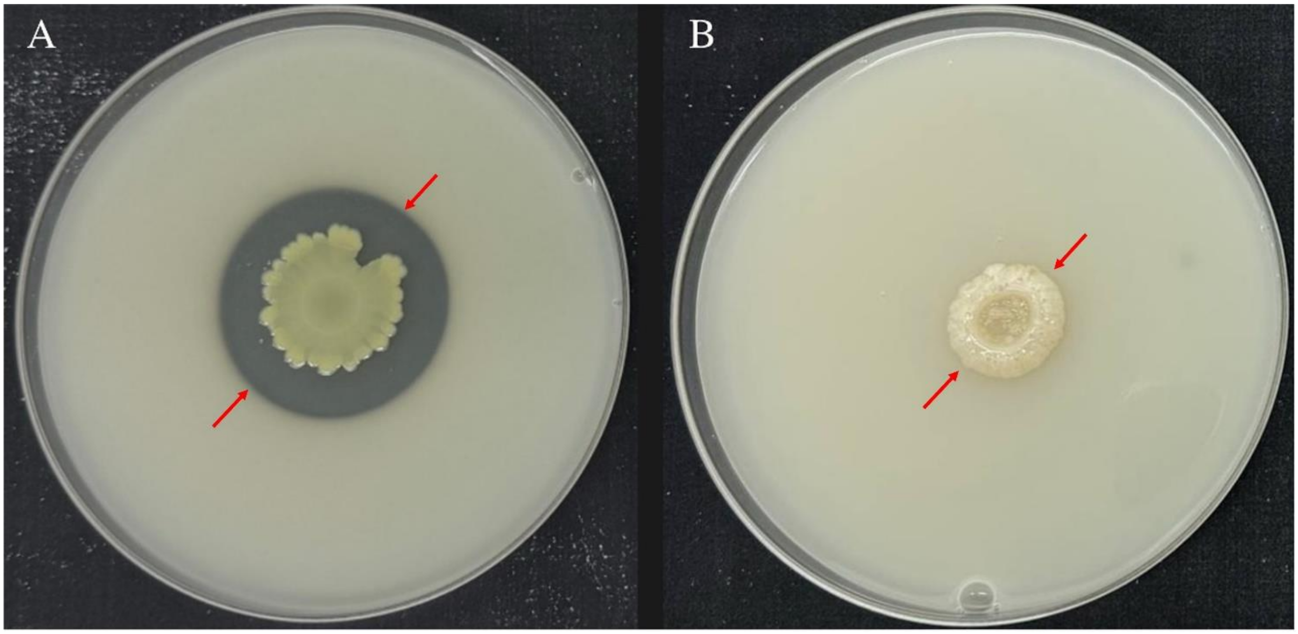

Supplementary Figure S1. Organic acid (OA) secretion of glyphosate-tolerant *Pseudomonas* sp. ADL-13 (A) and *Paenibacillus* sp. ZHS-9 (B). Red arrowheads indicate the outer boundary of the prominent halo for ADL-13 and the faint, localized clearing of putative OA secretion for ZHS-9.

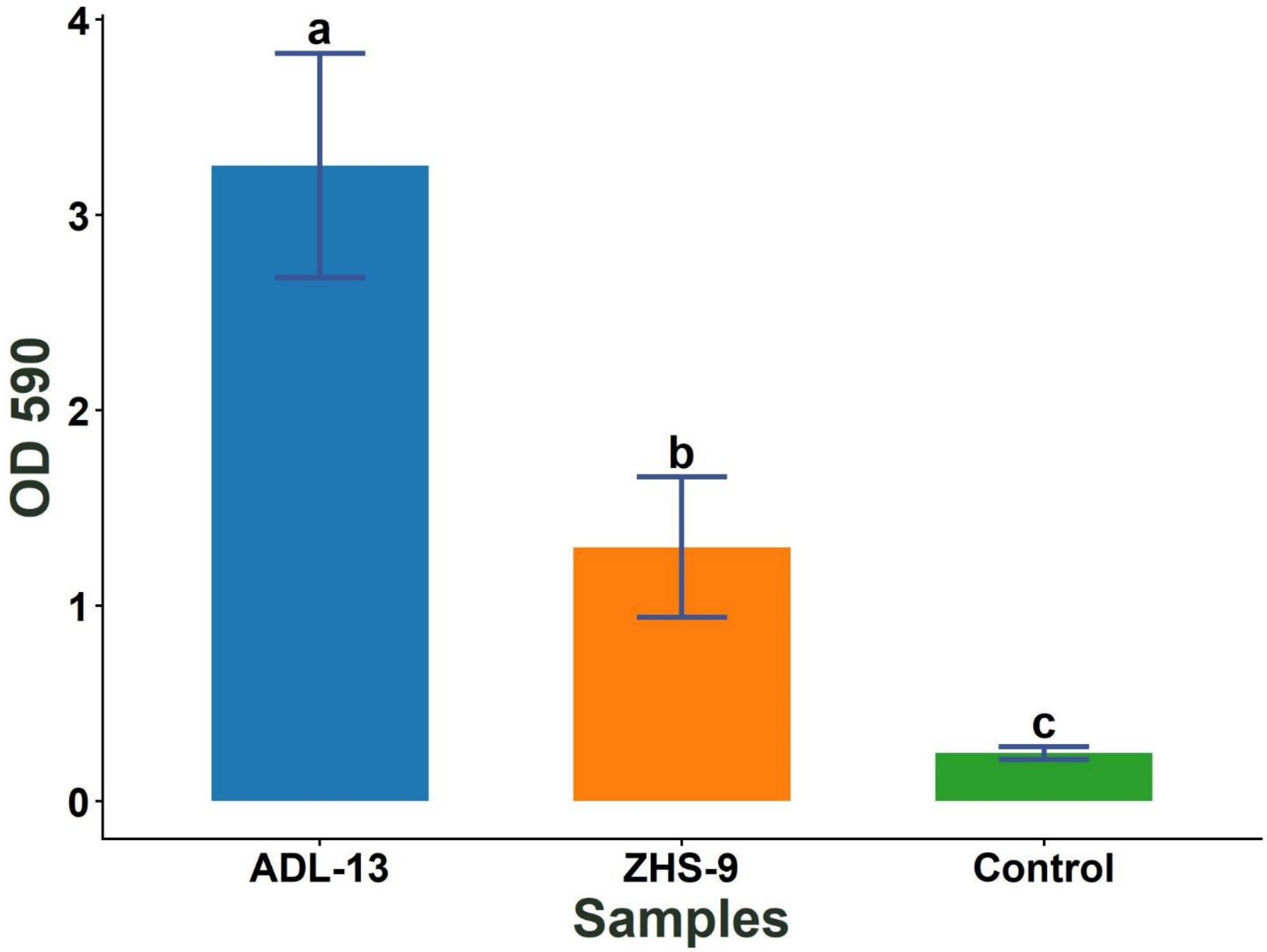

Supplementary Figure S2. Biofilm formation of glyphosate-tolerant *Pseudomonas* sp. ADL-13 and *Paenibacillus* sp. ZHS-9. Higher OD_{590} values relative to the non-inoculated control indicated greater biofilm-forming capacity under the assay conditions. Different letters indicate significant differences according to one-way ANOVA followed by Tukey’s HSD test ($p<0.05$). Bars represent the mean of six replicates (**n = 6**), and error bars represent standard deviations (SD).

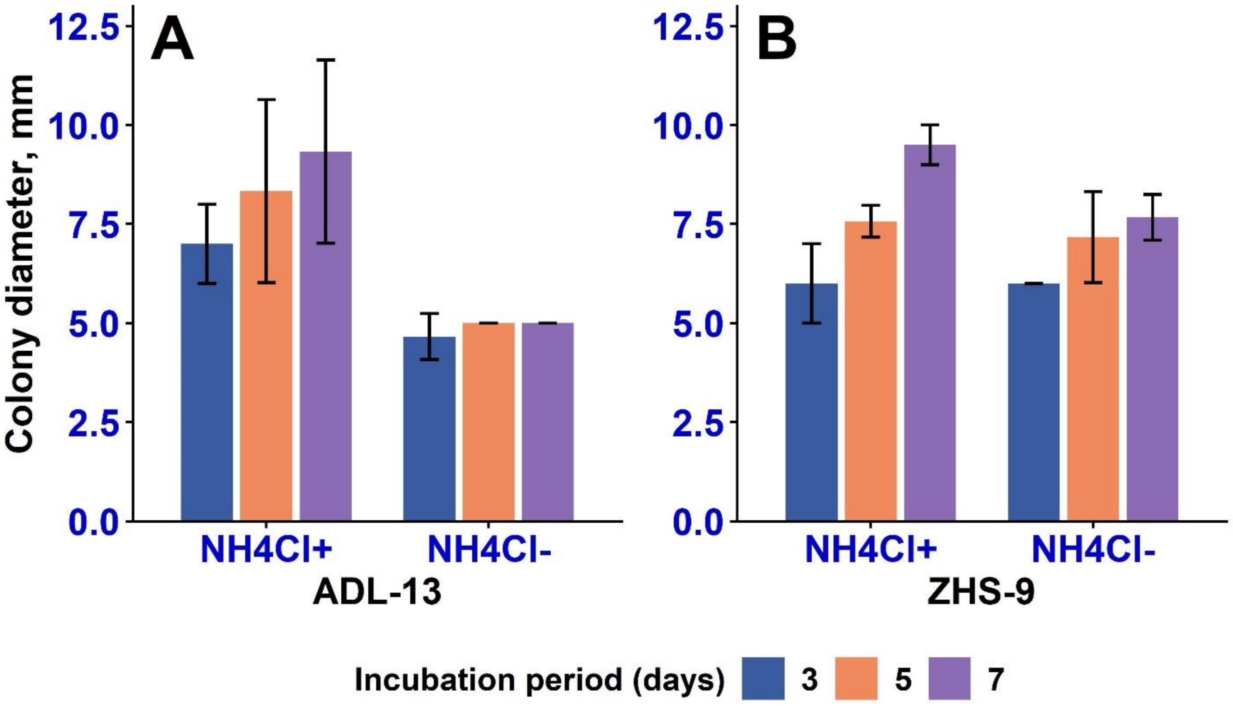

Supplementary Figure S3. The growth of glyphosate-tolerant *Pseudomonas* sp. ADL-13 (A) and *Paenibacillus* sp. ZHS-9 (B) on nitrogen-free Ashby's medium (NH_4Cl-) and Ashby's medium supplied with 0.27 g/L NH_4Cl (NH_4Cl+) as the control. Growth on nitrogen-free medium as an indicator of putative N-fixing potential.

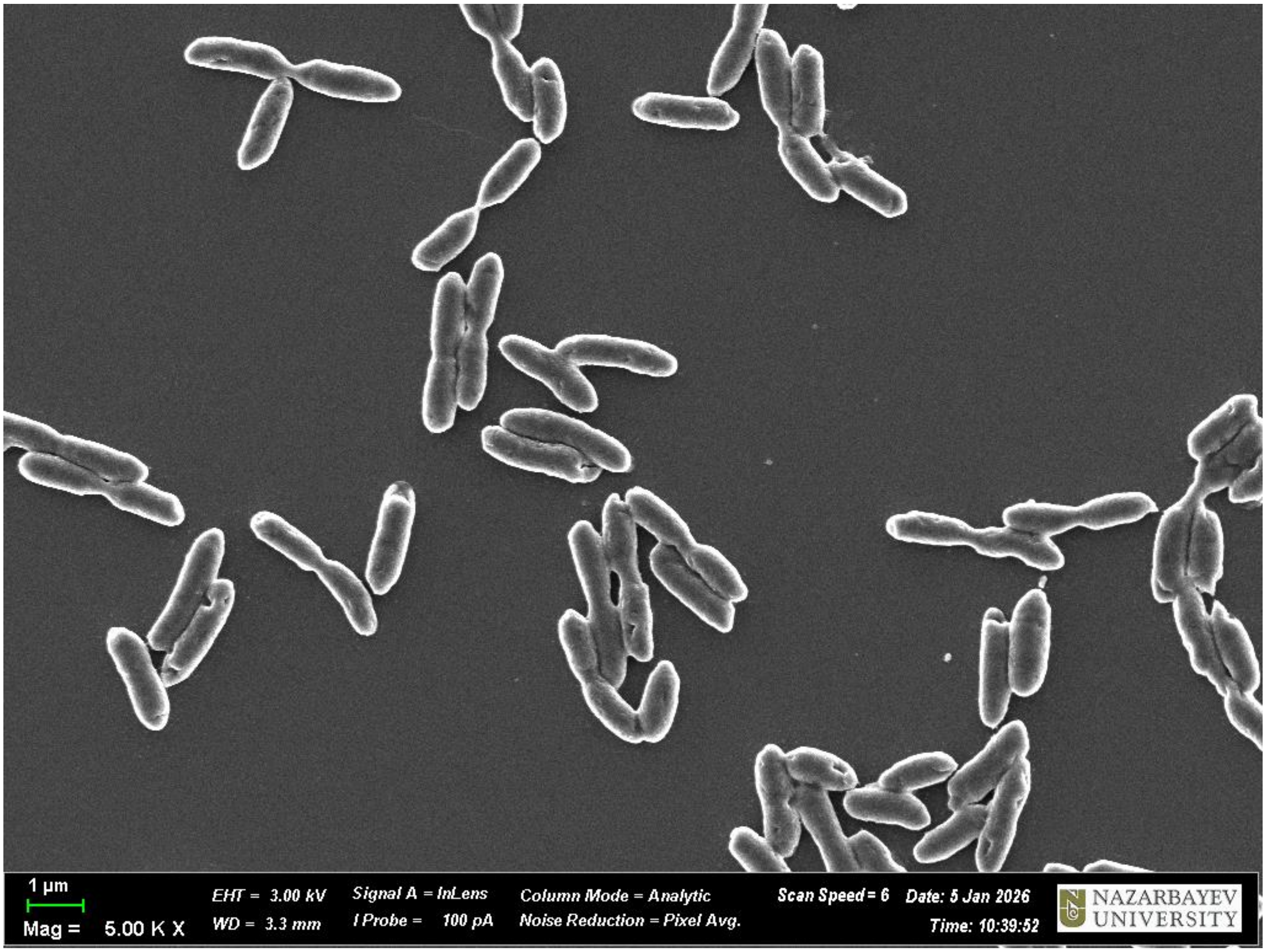

Supplementary Figure S4. SEM image of *Pseudomonas* sp. ADL-13 strain as shown in Figure 8A (**uncropped original image**).

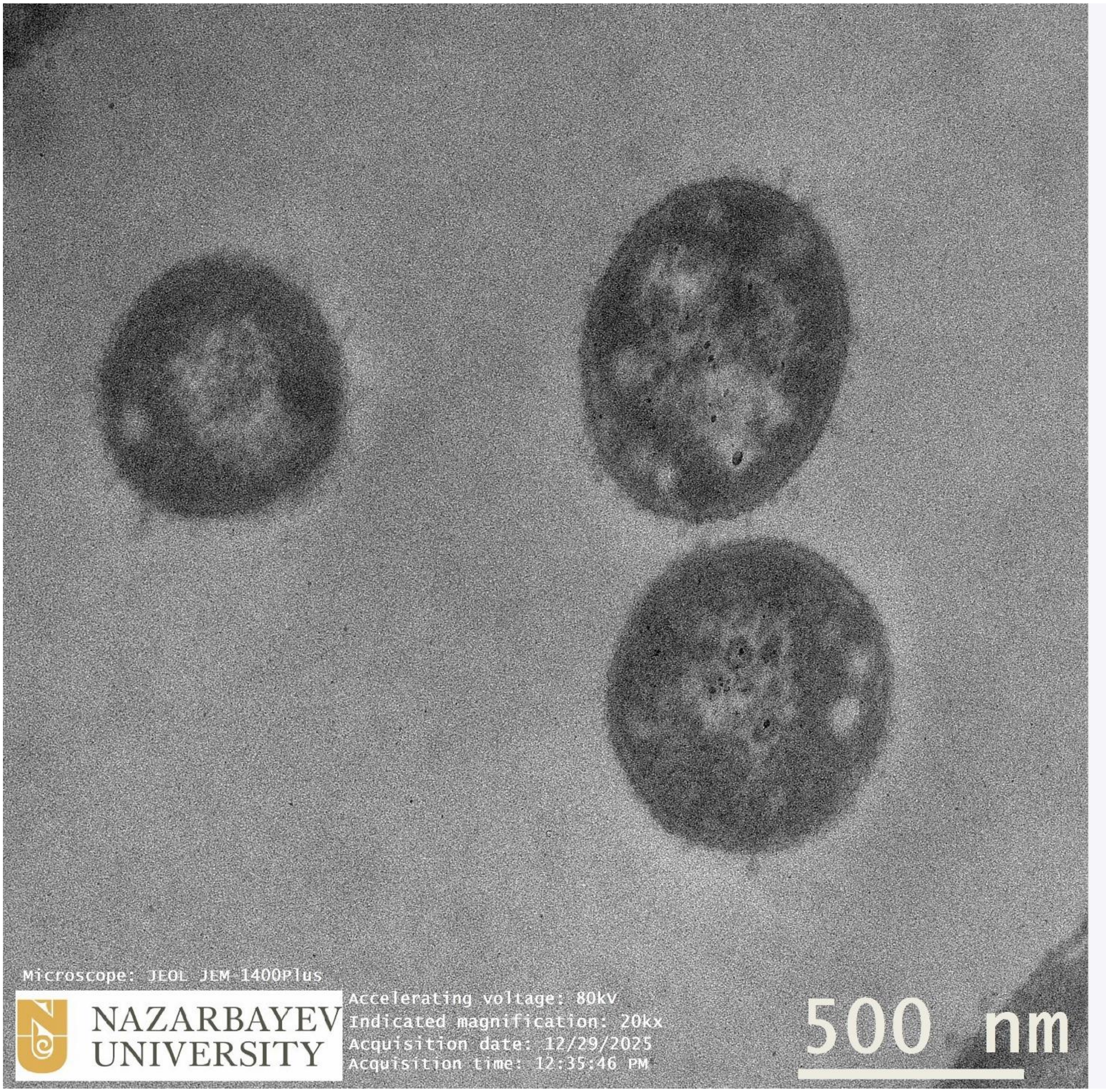

Supplementary Figure S5. TEM image of *Pseudomonas* sp. ADL-13 strain as shown in Figure 8B (**uncropped original image**).

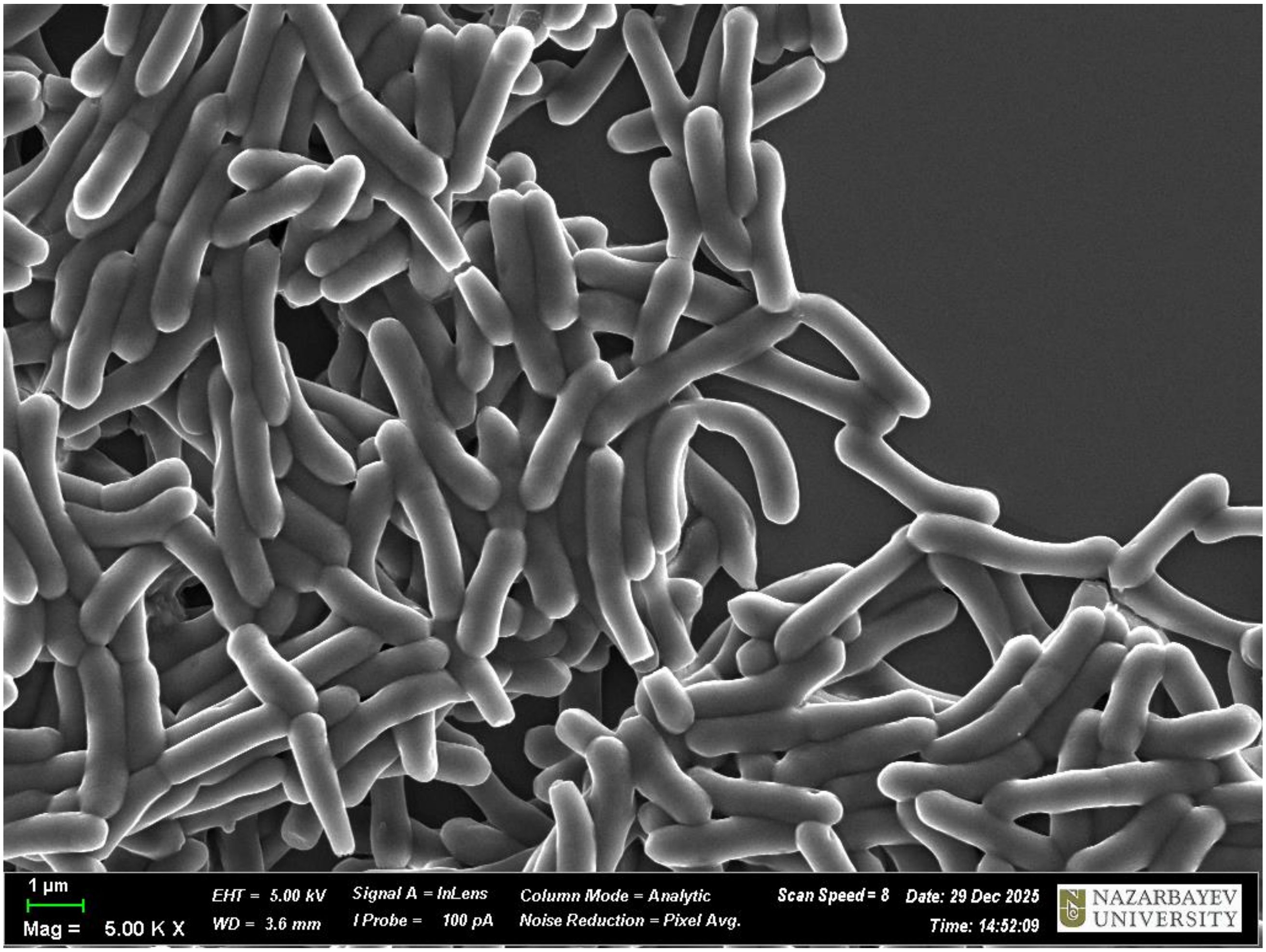

Supplementary Figure S6. SEM image of *Paenibacillus* sp. ZHS-9 strain as shown in Figure 8C (**uncropped original image**).

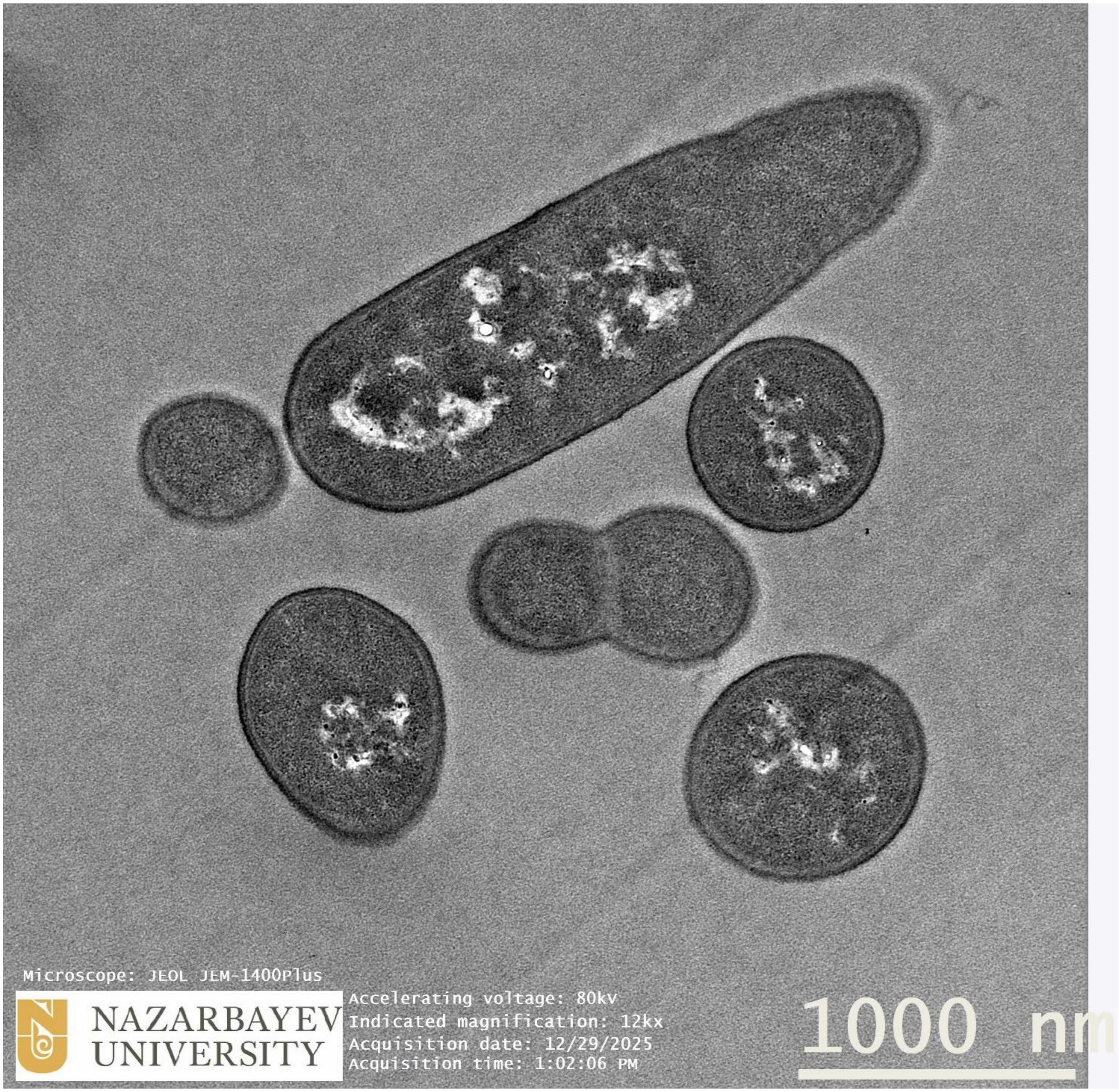

Supplementary Figure S7. TEM image of *Paenibacillus* sp. ZHS-9 strain as shown in Figure 8D (**uncropped original image**).
